# OsGSK2‐OsTCP19 Module Integrates Nitrogen and Brassinosteroid Signaling to Regulate Nitrogen Utilization and Root Growth in Rice

**DOI:** 10.1002/advs.74856

**Published:** 2026-03-15

**Authors:** Yongqiang Liu, Weiwei Li, Xiaohui Ma, Xiaohan Wang, Yarong Shi, Xiahe Huang, Xinran Li, Yingchun Wang, Hongning Tong, Bin Hu, Wenbin Zhou, Chengcai Chu

**Affiliations:** ^1^ Institute of Genetics and Developmental Biology Chinese Academy of Sciences Beijing China; ^2^ State Key Laboratory of Crop Gene Resources and Breeding National Key Facility for Crop Gene Resources and Genetic Improvement Institute of Crop Sciences Chinese Academy of Agricultural Sciences Beijing China; ^3^ Key Laboratory for Enhancing Resource Use Efficiency of Crops in South China Ministry of Agriculture and Rural Affairs South China Agricultural University Guangzhou China; ^4^ Key Laboratory of Plant Molecular Physiology Institute of Botany Chinese Academy of Sciences Beijing China; ^5^ State Key Laboratory of Forage Breeding‐by‐Design and Utilization Institute of Botany Chinese Academy of Sciences Beijing China

## Abstract

Nitrogen (N) and brassinosteroids (BRs) are key regulators of plant developmental plasticity in fluctuating nutrient environments, yet the direct molecular link coordinating these two signaling pathways remains elusive. Here, we report a synergistic interplay between nitrate and BR signaling that governs N utilization and lateral root development in rice (*Oryza sativa* L.). Nitrate activates BR response in a dose‐dependent manner, with maximal induction at 2.5 mM nitrate. Conversely, activated BR signaling enhances nitrate signaling and lateral root elongation, which strictly depends on the protein level of OsTCP19, a negative regulator of root growth. Nitrate‐induced BR signaling promotes OsTCP19 degradation within 2–4 h, while the BR signaling core kinase OsGSK2 interacts with and phosphorylates OsTCP19 at Ser141 and Thr289 to stabilize its protein. OsTCP19 directly binds to the promoters of nitrate‐responsive genes (e.g., *OsNRT2.4*, *OsNADH‐GOGAT1*, *OsASN1*) and root development genes (e.g., *OsIAA3*, *OsPIN1b*, *OsARF19*) to negatively regulate N responses and lateral root growth, respectively. Together, the OsGSK2‐OsTCP19 module establishes a direct molecular link between nitrate and BR signaling, coordinating N utilization and root plasticity in rice.

## Graphical abstract

OsGSK2‐OsTCP19 module regulating nitrate response and lateral root (LR) development. Low‐nitrate condition results in reduced BR response, accumulation of OsGSK2 and phosphorylated OsTCP19, which suppresses the expression of nitrate‐responsive genes and LR‐development genes and impairs rice growth. While high‐nitrate level results in high BR response and decreased OsGSK2 level, this diminishes the phosphorylated OsTCP19 level, leading to its degradation and alleviating the inhibition of nitrate response and LR elongation, thereby promoting the growth and development of rice.

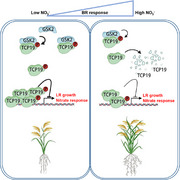

## 1 Introduction

Plants have evolved sophisticated regulatory networks for adaptation to dynamic nutrient availability. Nitrogen (N), a limiting macronutrient, shapes plant architecture primarily via ammonium (NH_4_^+^) and nitrate (NO_3_^−^) in soils. Beyond its nutritional role, nitrate acts as a signal to trigger genome‐wide transcriptional reprogramming, enabling adaptive phenotypic plasticity. Nitrate signaling pathways, such as the AtNRT1.1/CHL1‐Ca^2+^‐AtCPKs‐AtNLP7 cascade in *Arabidopsis* and the dual regulatory network of OsNRT1.1B‐OsNBIP1‐OsSPX4‐OsNLP3 and OsNRT1.1B‐OsCNGC14/16‐Ca^2+^‐OsNLP3 in rice, are well‐characterized centering on the transceptor NRT1.1 and core transcription factor NIN‐like proteins (NLPs) [1, 2, 3]. These pathways activate nitrate‐responsive genes involved in N utilization and plant development, with roots, the primary nutrient‐sensing organs, exhibiting plastic lateral root elongation positively correlated with nitrate concentration [4].

Brassinosteroids (BRs) are steroid phytohormones essential for plant growth and development, regulating morphogenesis, nutrient utilization, and stress responses [5]. Active BRs (e.g., brassinolide, BL) are synthesized in different tissues/organs via multi‐step pathways, with key genes identified in *Arabidopsis* (e.g., *DWARF1*, *DET2*) and in rice (e.g., *BRD1*, *BRD2*, *D2*, *D11*, and *OsDWARF4*). In the canonical BR signaling pathway, BR binds to the plasma membrane receptor BRI1 and co‐receptor BAK1, initiating a phosphorylation cascade that inactivates GSK3/SHAGGY‐like kinases (GSKs), core inhibitory components, to release downstream transcription factors (e.g., BZR1/BES1) to modulate BR‐responsive genes [6, 7]. BRs exert pleiotropic effects on plant architecture (plant height, tiller/branch number, tiller and leaf angle, grain size, and root length) in a dose‐dependent manner: both deficiency and excess inhibit growth [8], highlighting the need for precise spatiotemporal regulation.

External N availability modulates endogenous BR biosynthesis and signaling in spatiotemporal‐ and dose‐dependent manner [9, 10, 11]. In *Arabidopsis*, mild N‐deficiency upregulates *DWARF1* (*DWF1*) and *BSK3* to activate BR biosynthesis/signaling and promote root foraging [12, 13]. In rice, high N activates BR response via the OsLBDs‐OsTCP19‐DLT module to promote tillering [14], and high ammonium enhances BR biosynthesis to inhibit seminal root elongation via *miR444*‐mediated suppression of *OsMADS* genes [15]. BR signaling components (e.g., BES1, HBI1, RAVL1) also regulate N uptake and utilization [16, 17]. However, the molecular mechanism underlying the direct interplay between nitrate and BR signaling, particularly their coordination of N utilization and root development, remains unclear.

Here, we demonstrate that nitrate activates BR signaling in a dose‐dependent manner, and BR signaling in turn modulates nitrate response via the OsGSK2‐OsTCP19 module. OsTCP19 acts as a negative regulator downstream of OsGSK2, integrating nitrate and BR signals to control N utilization and lateral root growth. Our findings uncover a conserved regulatory axis that links nutrient signaling and hormone response, with implications for improving N use efficiency (NUE) in rice.

## 2 Results

### 2.1 N Activates BR Signaling in a Dose‐dependent Manner to Promote Rice Growth

**FIGURE 1:**
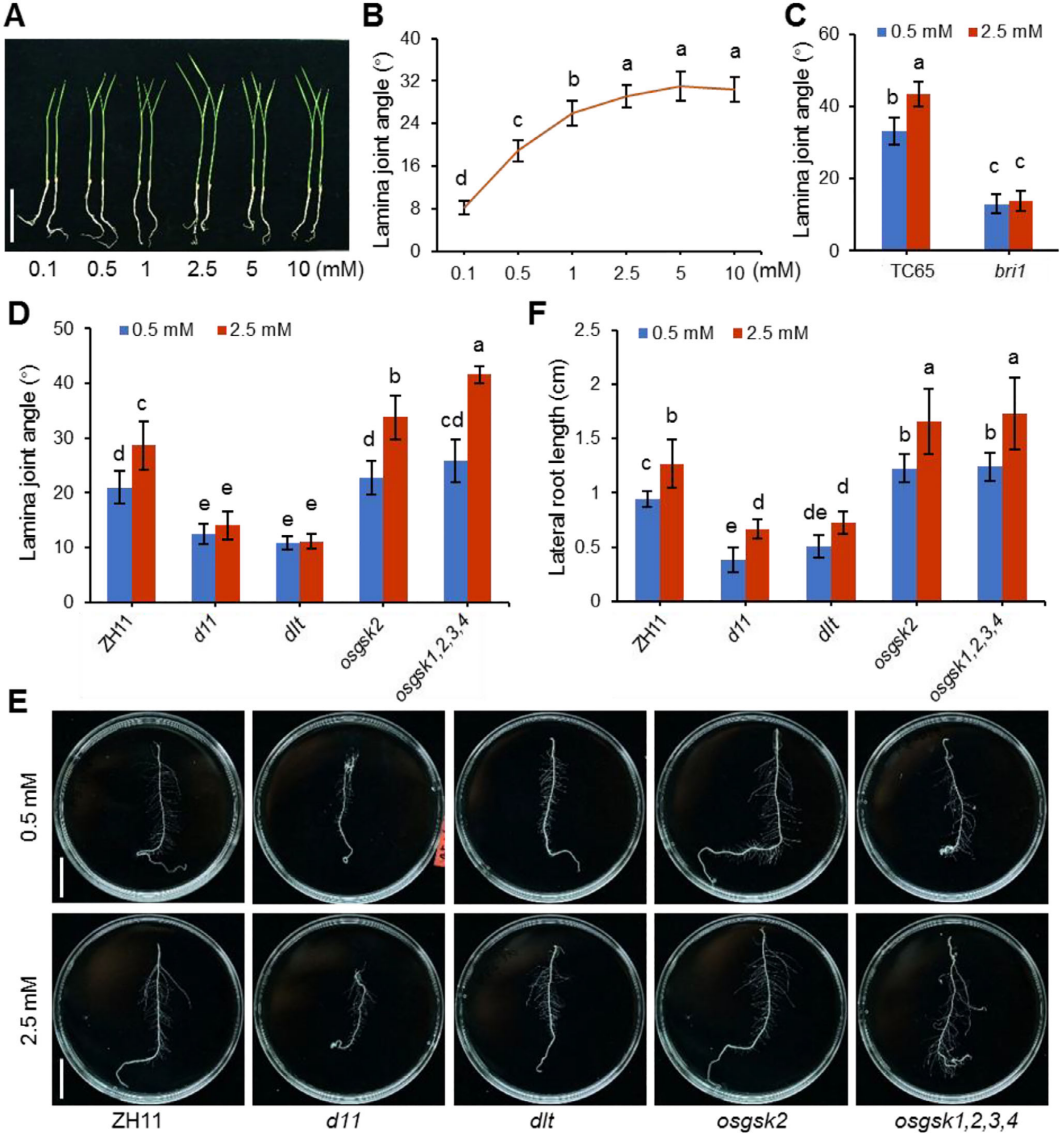
Nitrate activates BR response in a dose‐dependent manner. (A) Phenotype of rice seedlings grown under different nitrate concentrations (0.1, 0.5, 1, 2.5, 5, and 10 mm). Wild‐type Zhonghua 11 (ZH11) was used for hydroponic culture. Bar = 8 cm. (B) Lamina joint angle of rice seedlings grown under different nitrate concentrations. (C) Lamina joint angle of BR‐insensitive mutant *bri1* and its corresponding wild‐type Taichung 65 (TC65) to low (0.5 mm) and high (2.5 mm) nitrate. (D) Lamina joint angle of BR‐deficient (*d11*), BR‐insensitive (*dlt*), and BR‐hypersensitive (*osgsk2* and *osgsk1,2,3,4*) mutants (ZH11 background) under low‐ and high‐nitrate conditions. (E) Primary root phenotype of ZH11 and BR‐related mutants. Seminal roots of 10‐day‐old seedlings were sampled. Bars = 2 cm. (F) Lateral root length of ZH11 and BR‐related mutants. For (B‐D and F) values are mean ± s.d. (*n* = 12 plants), different letters indicate significant differences (*P* < 0.05, one‐way ANOVA, Tukey's HSD test).

Lamina joint angle is a well‐established index for BR sensitivity in rice [18, 19]. To investigate the physiological effect of N on BR responses, we grew rice seedlings under varying nitrate concentrations (0.1–10 mm) and measured lamina joint inclination. Nitrate treatment led to increased lamina joint angle in a dose‐dependent manner, peaking at 2.5 mm nitrate, with no further induction at higher concentrations (5–10 mm) (Figure 1A,B), indicating that N activates BR response within a physiological concentration range.

To confirm the involvement of BR biosynthesis or signaling, we tested BR‐related mutants under low (0.5 mm) and high (2.5 mm) nitrate levels. Both BR‐deficient (*d11*) and BR‐insensitive (*bri1*, *dlt*) mutants showed attenuated responses to high nitrate, while BR‐hypersensitive mutants (*osgsk2*, *osgsk1,2,3,4*) exhibited enhanced lamina joint angle (Figure 1C,D). This indicates that nitrate activates BR responses via BR biosynthesis and signaling pathways.

High nitrate‐promoted seedling growth was impaired in BR‐deficient/insensitive mutants but enhanced in BR‐hypersensitive mutants (Figure S1A–C). As nitrate concentrations increased, lateral root and seminal root lengths were significantly elevated in WT, *osgsk2*, and *osgsk1,2,3,4* plants, with the latter two mutants showing stronger induction (Figure 1E,F). In contrast, nitrate‐mediated stimulation was largely abrogated in BR‐deficient/insensitive mutants (Figure 1E,F), and lateral root density followed a similar trend (Figure S1D). Notably, while *d11* showed a weakened response to nitrate, nitrate‐induced biomass accumulation and root development were almost abolished in *dlt*, implying that BR signaling plays a more prominent role in mediating these nitrate responses (Figure 1F; Figure S1C). These data demonstrate that nitrate promotes lateral root growth in rice by activating the BR signaling pathway.

### 2.2 BR Signaling Enhances Nitrate Response and N Utilization in an N‐level‐dependent Manner

**FIGURE 2:**
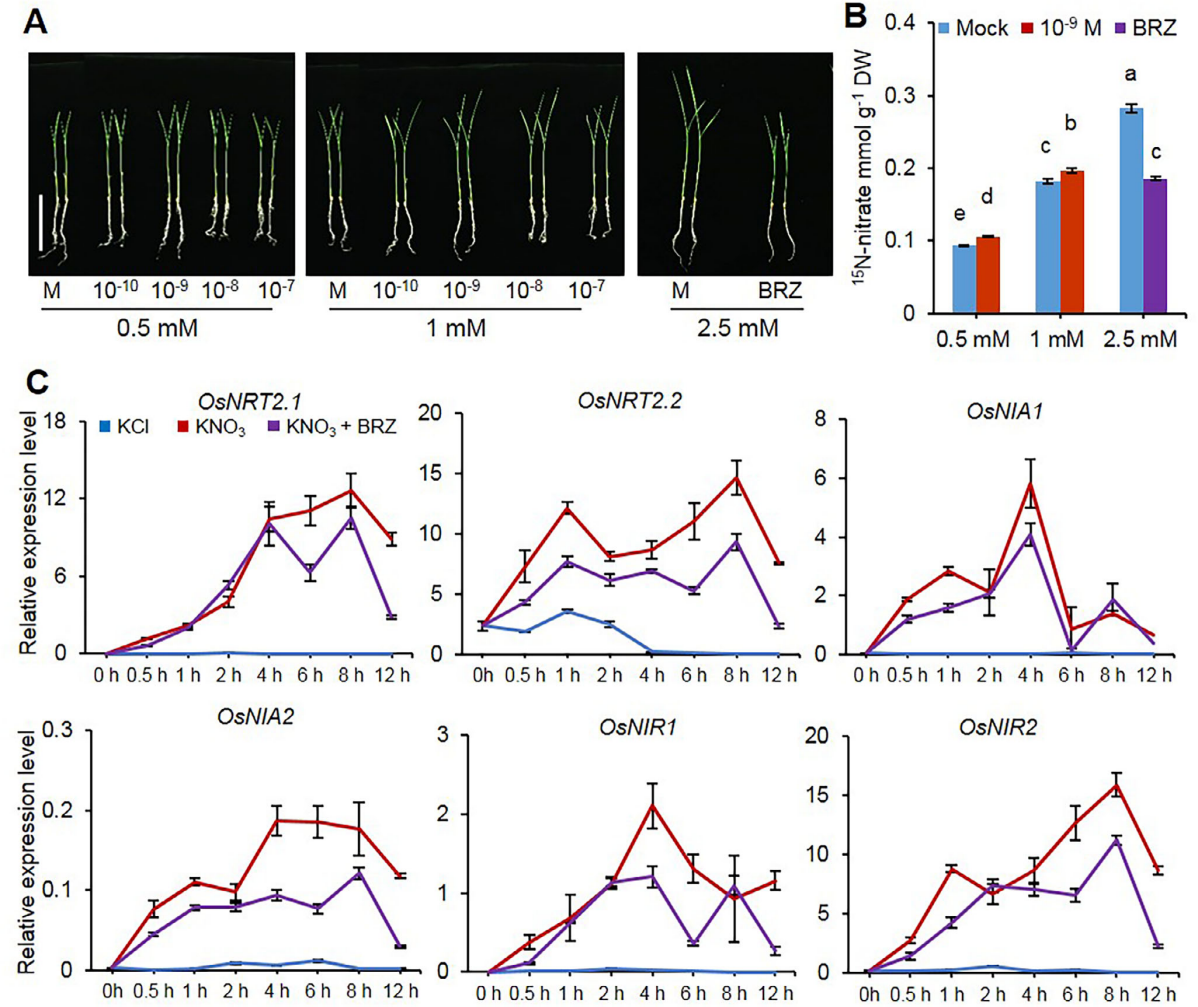
BR regulates nitrate signaling and utilization. (A) Phenotype of ZH11 seedlings treated with 0 (mock, M), 10^−10^, 10^−9^, 10^−8^, and 10^−7^ m BL under 0.5–2.5 mm nitrate. Bar = 8 cm. (B) ^15^N‐nitrate accumulation in seedlings grown under different nitrate concentrations treated with or without 10^−9^ m BL and 5 µm brassinazole (BRZ). DW, dry weight. Data are mean ± s.d. (*n* = 3 biological replicates). Different letters indicate significant differences (*P* < 0.05, one‐way ANOVA, Tukey's HSD test). (C) Expression of nitrate‐responsive genes in the roots of ZH11 plants treated with or without 5 µm BRZ. KCl as negative control. Data are means ± s.d. (*n* = 3 biological replicates).

We next tested whether enhanced BR signaling modulates nitrate signaling. Rice seedlings grown under low (0.5 mm) and moderate (1 mm) nitrate were treated with different concentrations of brassinolide (BL, active BRs). Lamina joint angle showed a positive dose‐response to BL: seedlings treated with 10^−8^–10^−7^ m BL exhibited enhanced lamina inclination under both nitrate conditions (Figure 2A; Figure S2A). Notably, at concentrations ranging from 10^−10^ to 10^−8^ M, BL stimulated the growth of shoots and roots under low and moderate nitrate levels (Figure 2A; Figures S2B, S3A, and S3B). In contrast, 10^−7^ m BL had little effect on shoots but strongly inhibited primary and lateral root elongation (though total root number increased) (Figure S3A–C), indicating distinct BR responsiveness across different plant organs.

High BL (10^−8^–10^−7^ m) exerted different effects under moderate than low nitrate conditions (Figure S2A,B), suggesting BR function is likely dependent on exogenous N availability. Specifically, 10^−10^ and 10^−9^ m BL significantly increased lateral root length under both low and moderate N. Conversely, treatment with brassinazole (BRZ), a specific inhibitor of BR synthesis, abrogated the growth‐promoting effects of high nitrate (Figure 2A,B; Figures S2 and S3).

To explore the molecular basis of BR‐nitrate crosstalk in regulating N utilization, we pre‐treated seedlings with BRZ before nitrate provision. BRZ largely repressed the induction of canonical nitrate‐responsive genes (*OsNRT2.1*, *OsNRT2.2*, *OsNIA1*, *OsNIA2*, *OsNIR1*, and *OsNIR2*) within 12 h and impaired N uptake under high nitrate (Figure 2B,C). While BR treatment remarkably stimulates the induction of these genes (Figure S4A). Correspondingly, nitrate response and nitrate uptake were severely impaired in *d11* but activated in *osgsk2* plants (Figure S4B,C). These findings demonstrate that BR is required for efficient nitrate signaling and N utilization.

### 2.3 OsTCP19 Mediates the Synergistic Interaction Between Nitrate and BR Signaling

**FIGURE 3:**
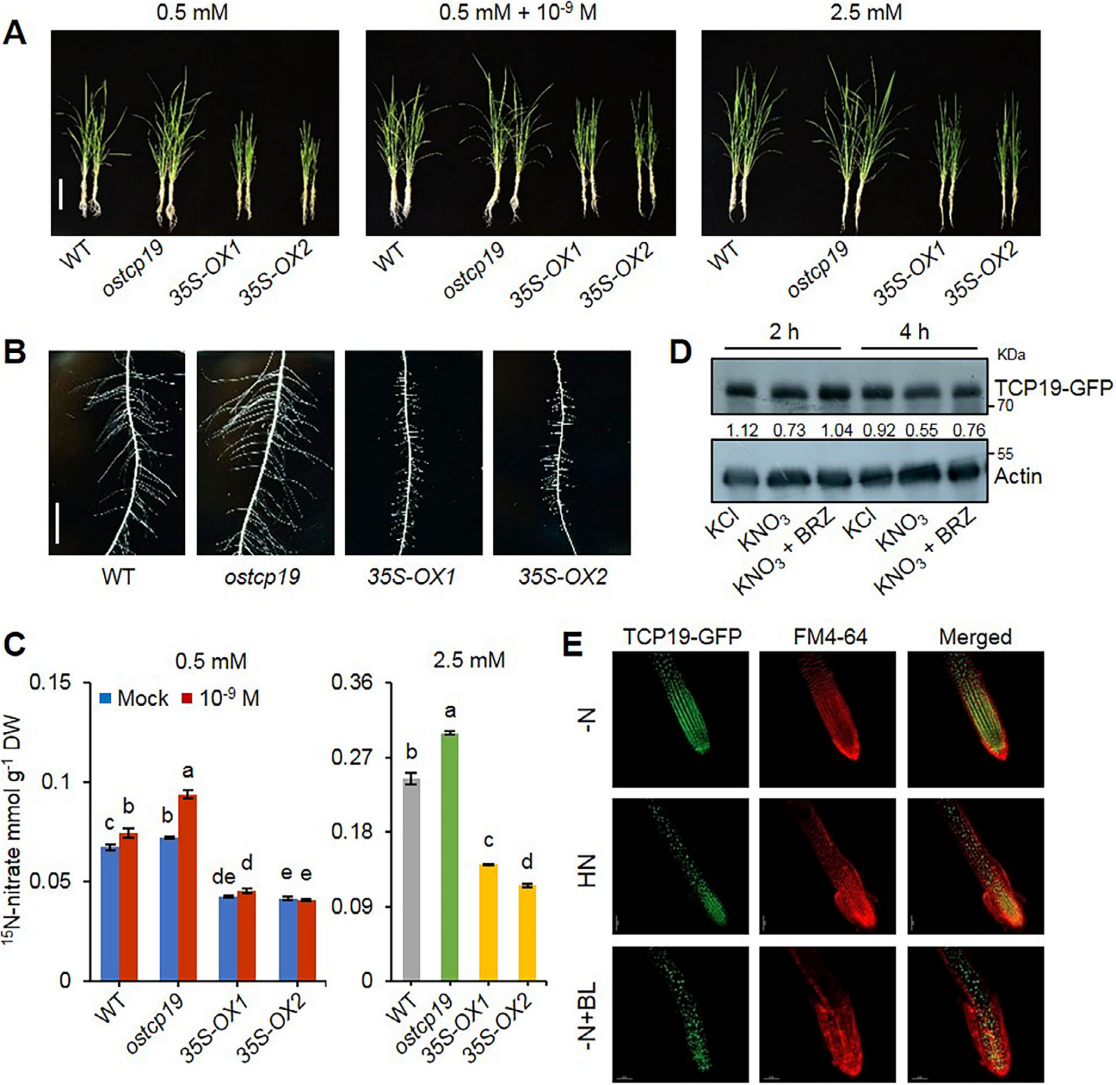
OsTCP19 mediates crosstalk between nitrate and BR signaling. (A) Phenotype of WT, *ostcp19*, and two *OsTCP19*‐overexpressing lines (*35S‐OX1* and *35S‐OX2*) grown under low nitrate ± BL (10^−9^ m) or high nitrate. Bar = 12 cm. (B) Lateral root phenotype of WT, *ostcp19* mutant, *35S‐OX1*, and *35S‐OX2* plants grown under high nitrate. Bar = 1 cm. (C) ^15^N‐nitrate accumulation in WT and *OsTCP19*‐related genetic lines grown under different nitrate concentrations ± BL (10^−9^ m). DW, dry weight. Data are mean ± s.d. (n = 3 biological replicates). Different letters indicate significant differences (*P* < 0.01, one‐way ANOVA, Tukey's HSD test). (D) Protein level of OsTCP19‐GFP in *35S‐OsTCP19‐GFP* transgenic plants after nitrate induction ± BRZ treatment. Actin as loading control. Each band was quantified by ImageJ. (E) Confocal images of OsTCP19‐GFP in lateral root tips under ‐N (no N), high nitrate (HN, 2.5 mm), and ‐N + BL. FM4‐64 as plasma membrane maker. Bars = 50 µm.

Our previous study revealed that high N (particularly nitrate) represses *OsTCP19* transcription to regulate rice tillering in long‐term cultivation [14]. Here, we investigated OsTCP19's role in short‐term nitrate response. Under high nitrate, the *ostcp19* mutant exhibited increased leaf angle and enhanced growth compared to WT, while *OsTCP19*‐overexpressing lines showed no significant response (Figure 3A; Figure S5A–C). Lateral root length in *ostcp19* was more sensitive to nitrate than WT, while lateral root elongation was severely inhibited in *OsTCP19*‐overexpressing lines across all nitrate concentrations (Figure 3B; Figure S5D), indicating *OsTCP19* acts as a negative regulator of N‐triggered BR signaling.

We next tested whether BR‐mediated regulation of N utilization depends on *OsTCP19*. BL treatment increased nitrate uptake in both WT and *ostcp19* under low N, with the mutant showing heightened BR sensitivity (Figure 3C). In contrast, *OsTCP19* overexpression nearly abrogated BR's effect on nitrate accumulation (Figure 3C). Lamina joint angle, shoot growth, and lateral root development also responded similarly to BL (Figure 3A,B; Figure S5B–D), confirming that *OsTCP19* is required for the synergistic interaction between nitrate and BR signaling.

*OsTCP19* transcription was unresponsive to short‐term nitrate or BRZ treatment (Figure S5E), so we focused on protein‐level regulation. High nitrate (5 mM) induced a marked reduction in OsTCP19 protein abundance at 2–4 h, an effect abrogated by BRZ co‐treatment (Figure 3D), indicating that endogenous BR is essential for nitrate‐induced OsTCP19 degradation. Subcellular localization analysis showed OsTCP19 is exclusively localized to the nucleus in cells spanning the root meristematic to elongation zones, with prominent expression in root tips (consistent with its role in regulating lateral root elongation) (Figure 3E). Fluorescent signals of OsTCP19 in root tips were attenuated by high nitrate or BL treatment, aligning with the immunoblot results (Figure 3D,E). These results demonstrate that nitrate‐activated BR signaling promotes OsTCP19 degradation, enabling OsTCP19 to negatively regulate nitrate utilization and lateral root development.

### 2.4 OsTCP19 Acts Downstream of OsGSK2 in the BR Signaling Pathway

**FIGURE 4:**
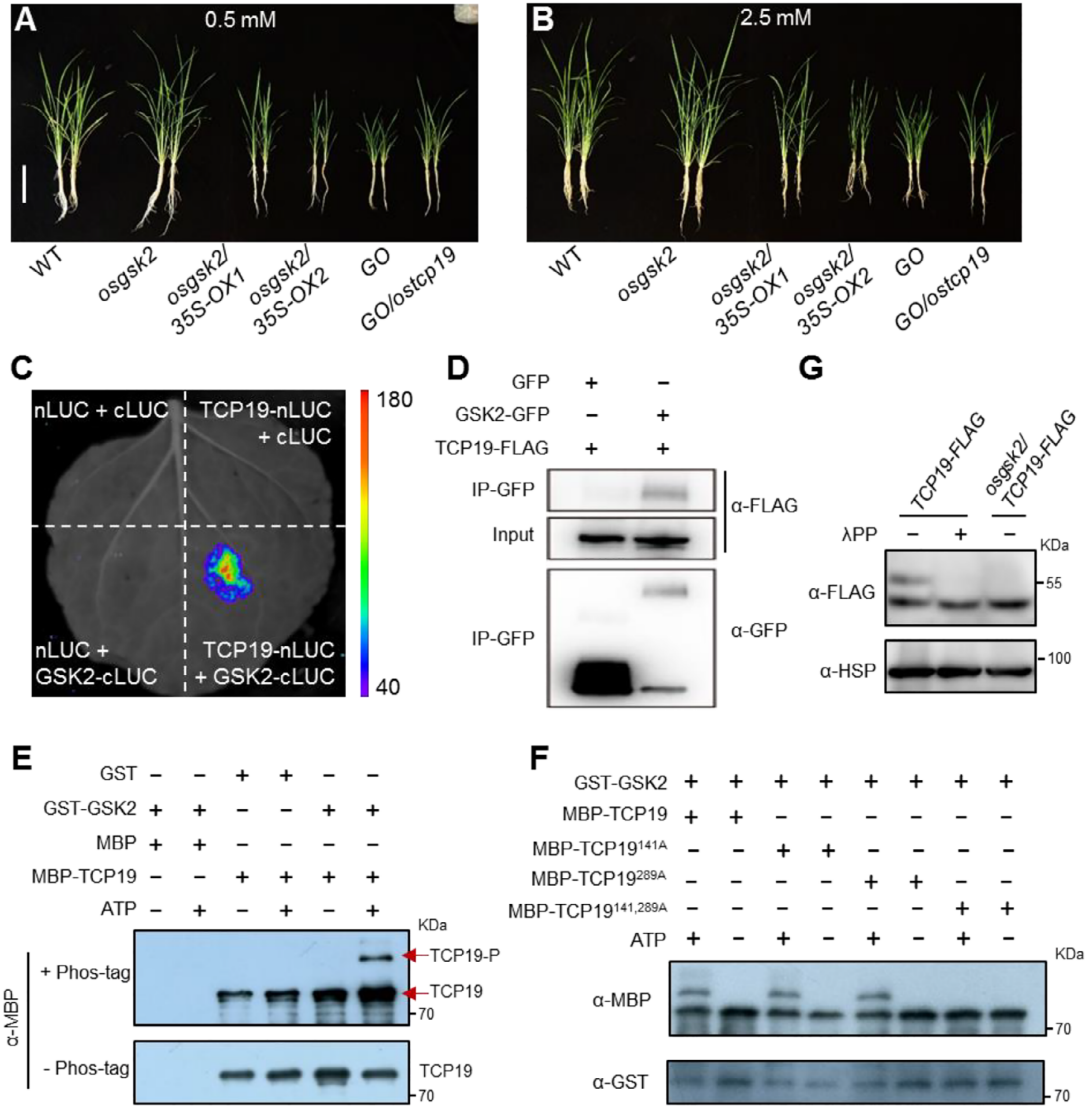
OsGSK2 interacts with and phosphorylates OsTCP19. (A,B) Phenotype of wild‐type (WT) ZH11, *osgsk2*, *OsTCP19*‐overexpressing lines under *osgsk2* background (*osgsk2/35S‐OX1* and *osgsk2/35S‐OX2*), GO, and GO/*ostcp19* plants grown under different low (0.5 mm) (A) and high nitrate (2.5 mm) (B) conditions. Bar = 12 cm. (C) LCI assay of OsGSK2 and OsTCP19 in tobacco leaves. (D) Co‐IP assay with OsGSK2‐GFP and OsTCP19‐FLAG co‐expressed in tobacco leaves. (E) OsGSK2 phosphorylates OsTCP19 in vitro. Different recombinant proteins were incubated for phosphorylation reaction, separated by normal or Phos‐tag SDS‐PAGE, and immunoblotted, respectively. (F) Mutation of OsTCP19^S141A, T289A^ abolishes the phosphorylation by OsGSK2. Single mutations of OsTCP19^141A^ and OsTCP19^289A^ as well as double mutation of OsTCP19^S141A, T289A^ were used for the kinase assay. (G) OsGSK2 phosphorylates OsTCP19 in vivo. OsTCP19‐FLAG overexpressing in ZH11 and *osgsk2* background were used. λPP (lambda protein phosphatase) treatment confirms phosphorylation‐dependent mobility shift. HSP was used as a loading control.

OsGSK2 is a central negative regulator of BR signaling in rice, modulating substrate stability or abundance via physical interaction [6]. Given OsTCP19's role in nitrate‐activated BR signaling, we hypothesized that OsGSK2 and OsTCP19 function in the same pathway. Genetic analysis showed that *OsTCP19*‐overexpressing lines (*35S‐OX1/2*) exhibit a BR‐deficient phenotype similar to *OsGSK2*‐overexpressing (*GO*) plants (Figures 3A and 4A). Crosses between *35S‐OX1/2* and *osgsk2* mutants (*osgsk2/35S‐OX1/2*) significantly suppressed high nitrate‐induced growth and lateral root elongation compared to *osgsk2* (Figure 4A,B; Figure S6A–C).

Conversely, *OsTCP19* knock‐out in *GO* plants (*GO*/*ostcp19*) partially rescued the nitrate‐insensitive phenotype of *GO* lines (Figure 4A,B; Figure S6A–C). Additionally, the enhanced BR sensitivity of *osgsk2* mutants (manifested by increased lateral root length) was abrogated in *osgsk2*/*35S‐OX1/2* plants, while BR sensitivity was moderately restored in *GO/ostcp19* double mutants compared to *GO* lines (Figure S6A–C). These genetic data establish that OsTCP19 acts downstream of OsGSK2.

Luciferase complementation imaging (LCI) assays in tobacco (*Nicotiana benthamiana*) leaves showed that a strong fluorescence signal when *OsGSK2* and *OsTCP19* were co‐expressed, indicating their direct interaction (Figure 4C). This interaction was further validated by co‐immunoprecipitation (co‐IP) assays, confirming that OsGSK2 associates with OsTCP19 in vivo (Figure 4D). In vitro kinase assays showed that co‐incubation of recombinant OsGSK2 and OsTCP19 proteins resulted in a distinct mobility shift of OsTCP19 in Phos‐tag SDS‐PAGE gels (which specifically retard phosphorylated proteins), indicating that OsTCP19 is phosphorylated by OsGSK2 in vitro (Figure 4E). Mass spectrometry analysis of phosphorylated OsTCP19 identified two phosphorylation sites: Ser141 (S141) and Thr289 (T289) (Figure S7). To validate that these residues are direct targets of OsGSK2, we generated a non‐phosphorylatable OsTCP19 mutant by substituting S141 and T289 with alanine (S141A and T289A). In vitro kinase assays showed that in comparison to two single mutants, the S141A/T289A double mutation completely abolished OsGSK2‐mediated phosphorylation of OsTCP19 (Figure 4F). Moreover, it was shown that the in vivo phosphorylation of OsTCP19 is dependent on OsGSK2 (Figure 4G). Together, these results demonstrate that OsGSK2 physically interacts with and phosphorylates OsTCP19 at S141 and T289, acting upstream of OsTCP19 in the BR signaling pathway.

### 2.5 Phosphorylation of OsTCP19 Stabilizes Its Protein and Enhances Its Functional Activity

**FIGURE 5:**
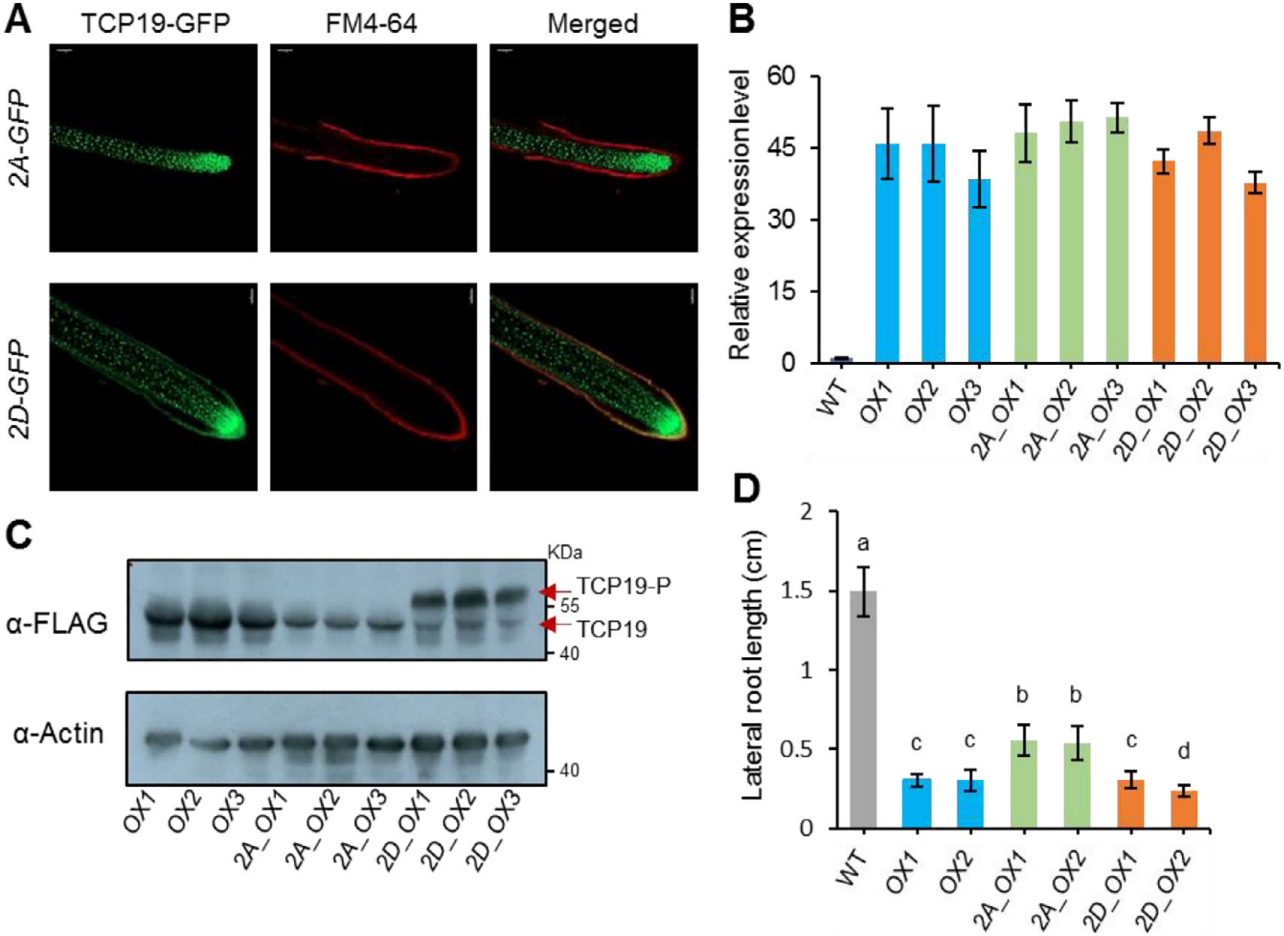
Phosphorylation of OsTCP19 stabilizes its protein level. (A) Confocal images of lateral root tips from rice seedlings expressing *OsTCP19^S141A, T289A^‐GFP* (2A‐GFP) and *OsTCP19^S141D, T289D^‐GFP* (2D‐GFP). FM4‐64 as plasma membrane maker. Bars = 50 µm. (B) Relative expression of *OsTCP19* variants in overexpression lines. Transcriptional level in WT was set as “1”. Values are the means ± s.d. (n = 3 biologically independent samples). (C) Protein level of OsTCP19 variants from transgenic lines of (B). Actin as loading control. (D) Lateral root length of ZH11 and different *OsTCP19*‐overexpressing lines under high nitrate. Data are mean ± s.d. (*n* ≥ 20 plants). Different letters indicate significant differences (*P* < 0.05, one‐way ANOVA, Tukey's HSD test).

To explore the functional consequences of OsTCP19 phosphorylation, we generated transgenic plants overexpressing three *OsTCP19* variants: wild‐type *OsTCP19*, the non‐phosphorylatable mutant *OsTCP19* ^S141A, T289A^ (S141 and T289 substituted with Alanine), and the phospho‐mimic mutant *OsTCP19* ^S141D, T289D^ (S141 and T289 replaced by Aspartic acid). Subcellular localization assay showed that all variants localize to the nucleus (Figures 3E and 5A), indicating that phosphorylation does not alter the subcellular localization of OsTCP19.

Immunoblot analysis revealed that the protein level of OsTCP19^S141A, T289A^ was remarkably lower than wild‐type OsTCP19, while the phospho‐mimetic OsTCP19^S141D, T289D^ variant exhibited identical abundance, despite comparable transgene expression (Figure 5B,C). Consistent with this, lateral root length was significantly greater in OsTCP19^S141A, T289A^‐overexpressing lines than in *OsTCP19* WT or *OsTCP19* ^S141D, T289D^‐overexpressing lines (Figure 5D), implying impaired biological function of the non‐phosphorylatable OsTCP19 variant. Additionally, OsTCP19^S141A, T289A^ showed obviously weakened protein stability than WT and *OsTCP19* ^S141D, T289D^ (Figure S8). These results demonstrate that OsTCP19 phosphorylation enhances protein stability and functional activity in rice seedlings.

### 2.6 OsTCP19 Directly Targets Nitrate‐responsive Genes to Negatively Regulate Nitrate Signaling

**FIGURE 6:**
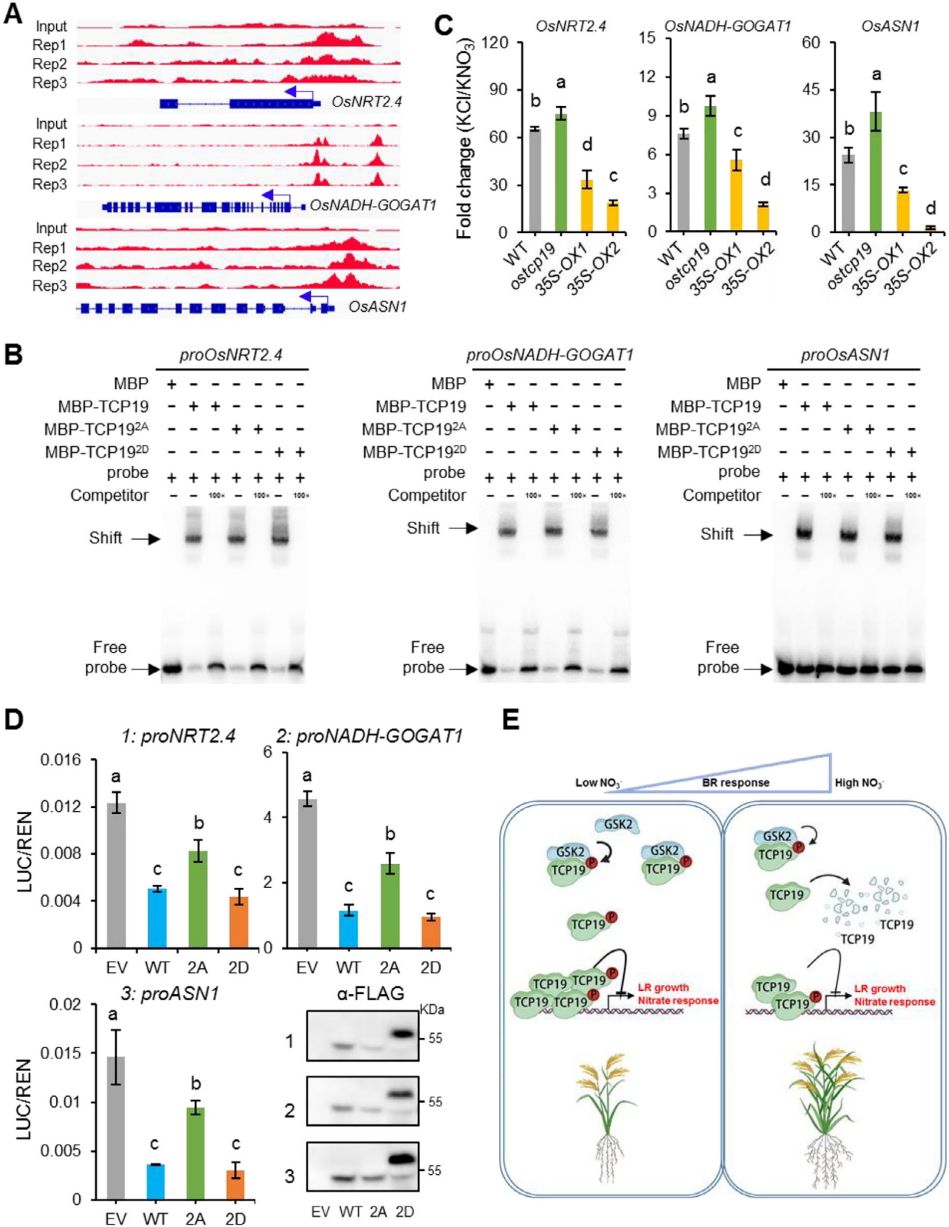
OsTCP19 targets nitrate‐responsive genes. (A) ChIP‐seq profiles of OsTCP19 binding to *OsNRT2.4*, *OsNADH‐GOGAT1*, and *OsASN1* promoters. (B) Different OsTCP19 variants (MBP‐TCP19, MBP‐TCP19^2A^, and MBP‐TCP19^2D^) can directly bind to the promoters of three nitrate‐responsive genes in electrophoretic mobility shift assay (EMSA). 100‐fold unlabeled wild‐type probes were used for competition. (C) Expression of nitrate‐responsive genes in the roots of WT, *ostcp19*, and *OsTCP19*‐overexpressing lines (5 mM KNO_3_, 6 h). Values are the means ± s.d. (*n* = 3 biological replicates). (D) Transcriptional repression effect of different OsTCP19 variants (co‐transformed with OsGSK2) to *luciferase* (*LUC*) reporter gene driven by promoters of nitrate‐responsive genes in rice protoplasts treated with 5 mM KNO_3_. EV, empty vector. WT, wild‐type OsTCP19. 2A, OsTCP19^S141A, T289A^. 2D, OsTCP19^S141D, T289D^. The protein levels of different OsTCP19 variants expressed in rice protoplasts were shown. Data are the means ± s.d. (*n* = 4 biological replicates). Different letters of (C) and (D) indicate significant differences (*P* < 0.05, one‐way ANOVA, Tukey's HSD test). (E) A proposed model of OsGSK2‐OsTCP19 module regulating nitrate response and lateral root (LR) development. Low‐nitrate condition results in reduced BR response, accumulation of OsGSK2 and phosphorylated OsTCP19, which suppresses the expression of nitrate‐responsive genes and LR‐development genes and impairs rice growth. While high‐nitrate level results in high BR response and decreased OsGSK2 level, this diminishes the phosphorylated OsTCP19 levels, leading to its degradation and alleviating the inhibition of nitrate response and LR elongation, thereby promoting the growth and development of rice. The picture was created in BioRender.

To investigate whether OsTCP19 directly modulates nitrate‐responsive genes, we analyzed our previously published ChIP‐seq data [20], and identified N‐related target genes (*OsNLP1*, *OsNLP3*, *OsNLP4*, *OsNRT2.4*, *OsNADH‐GOGAT1*, *OsFd‐GOGAT1*, *OsASN1*, and *OsAS2*) with fold enrichment > 2 and *P* < 0.001 (Figure 6A; Figure S9A). Expression profiling following nitrate induction showed *OsNRT2.4* and *OsASN1* are strongly induced by nitrate, while *OsNLP3*, *OsNLP4*, *OsNRT2.4*, *OsNADH‐GOGAT1*, and *OsFd‐GOGAT1* exhibit distinct nitrate responses (Figure S10). Electrophoretic mobility shift assay (EMSA) confirmed OsTCP19 directly binds to the GGNCCCAC motif in the promoters of *OsNRT2.4, OsNADH‐GOGAT1*, and *OsASN1* genes, and the interactions were competitively inhibited by unlabeled probes (Figure 6B). In addition, no differences in binding capacity were observed among the different OsTCP19 variants (Figure 6B).

Expression analysis showed nitrate‐induced transcription of these genes was enhanced in the *ostcp19* mutant but attenuated in *OsTCP19*‐overexpressing lines. Transcriptional activation assays in rice protoplasts demonstrated that co‐expression of *OsTCP19* significantly inhibits nitrate‐induced activity of reporter genes driven by these target promoters (Figure 6C; Figure S9B). Notably, the non‐phosphorylatable OsTCP19^S141A, T289A^ variant exhibited weak transcriptional repression, while the phospho‐mimetic OsTCP19^S141D, T289D^ variant showed strong repression, likely due to phosphorylation‐dependent differences in protein stability (Figure 6D). In addition, nitrate induction of these genes were triggered in *osgsk2* mutant but suppressed in *osgsk2/35‐OX1*, *osgsk2/35‐OX2*, *GO*, and *GO ostcp19* plants, further supporting the negative role of OsGSK2‐OsTCP19 module in nitrate signaling (Figure S11).

### 2.7 TCP19 Targets Root Development Genes to Negatively Regulate Lateral Root Length

To elucidate OsTCP19's role in lateral root development, we screened root development genes from OsTCP19 ChIP‐seq datasets with Gene Ontology annotations as root development regulation processes and identified 59 candidate genes (Table S1), including 7 auxin‐related genes (∼12% of total). This is consistent with the previous studies that both nitrate and BR affect root growth largely depending on auxin ([11]; Maghiaoui et al., 2020). Of them, 3 functionally characterized genes including *OsIAA3* (a root elongation repressor), *OsPIN1b* (auxin transporter), and *OsARF19* (auxin response factor) were reported to be closely related to lateral root development [21, 22, 23]. EMSA confirmed that OsTCP19 directly binds to the promoters of these genes, with no difference between its three variants (Figure S12A,B).

Expression analysis revealed that *OsIAA3* was down‐regulated in the *ostcp19* mutant but up‐regulated in *OsTCP19*‐overexpression lines, while *OsPIN1b* and *OsARF19* exhibited the opposite pattern (Figure S12C). Moreover, similar trend in the expression level of these genes was found in *osgsk2* and *GO* plants (Figure S12D). Notably, *OsIAA3*, *OsPIN1b*, and *OsARF19* were not transcriptionally induced by nitrate, suggesting their potential involvement in the long‐term nitrate response (Figure S13). These findings demonstrate that OsGSK2‐OsTCP19 module targets key root development genes to regulate lateral root growth, partially via auxin‐related pathways.

## 3 Discussion

### 3.1 Dose‐Dependent Synergism Between Nitrate and BR Signaling

N is a pivotal macronutrient that largely limits plant growth and crop yield. As an external environmental cue, N status is transduced into endogenous cellular signals to trigger plant developmental responses, which is tightly dependent on multiple phytohormones. BR acts as a key hormone that comprehensively regulates plant growth and development. Thus, deciphering the regulatory mechanisms underlying N‐BR crosstalk holds great promise for crop improvement in sustainable agriculture. Several studies have elucidated how N regulates BR biosynthesis and signaling components, as well as how BR modulates N uptake [12, 13, 15, 16, 17, 24]. However, a direct molecular link connecting N and BR signaling pathways remains scarce. In this study, we uncovered a dose‐dependent synergistic interplay between nitrate and BR signaling in regulating nitrate utilization and lateral root development in rice, which is mediated by the OsGSK2‐OsTCP19 module.

Both N and BR exert physiological effects in a dose‐dependent manner: either deficiency or excess inhibits plant growth. Our results showed that increasing nitrate supply positively activates BR responses, which plateau at ∼2.5 mM (Figure 1). Conversely, BR also positively regulates nitrate response, N utilization, and plant growth within a narrow concentration window (10^−10^–10^−8^ m BL) in rice seedlings (Figure 2). More importantly, BL treatment under low nitrate (0.5 mm) enhanced BR responses to levels comparable to moderate nitrate (1 mm), while blocking BR biosynthesis with BRZ under high nitrate (2.5 mm) induced BR‐deficient phenotypes similar to low N (Figure 2; Figures S2 and S3). Additionally, lower BL concentrations (10^−10^ and 10^−9^ m) exerted robust physiological effects under low N, while higher BL concentrations (10^−9^ and 10^−8^ m) were effective under moderate N (Figure 2; Figures S2 and S3). These observations indicate that N levels tightly control endogenous BR responses, which in turn regulate N utilization and plant growth to match external nutrient availability.

### 3.2 OsGSK2‐OsTCP19 Module Integrates Nitrate and BR Signaling in Rice

Previous studies have uncovered indirect crosstalk between N and BR signaling. In *Arabidopsis*, mild N deficiency induces *BSK3* to activate BR signaling and root foraging [13], while low nitrate upregulates *CALMODULIN‐LIKE‐38* (*CML38*) to inhibit BES1 phosphorylation and trigger BR responses [24]. In rice, we found that the OsLBD37/39‐OsTCP19‐DLT module links N and BR response to modulate rice tillering [14]. Here, we extend these findings by identifying the OsGSK2‐OsTCP19 module as a direct molecular bridge between nitrate and BR signaling (Figure 6E). OsGSK2, the core negative regulator of BR signaling, interacts with and phosphorylates OsTCP19 to stabilize its protein, while nitrate‐activated BR signaling promotes OsTCP19 degradation (Figures 3 and 4). OsTCP19 directly targets nitrate‐responsive genes and root development genes to negatively regulate N utilization and lateral root growth (Figures 6; Figure S12).

Recently, TaGSK2 was shown to phosphorylate TaNLP7 to inhibit N deficiency‐induced leaf senescence in wheat (Yang et al., 2025), suggesting a conserved mechanism whereby GSKs modulate NLP activity via phosphorylation. Future studies should explore the molecular interactions among OsGSK2, OsTCP19, and OsNLP3, as well as the crosstalk of upstream components between nitrate signaling (e.g., NRT1.1‐Ca^2+^ influx) and BR signaling (e.g., phosphorylation/dephosphorylation cascades).

### 3.3 OsTCP19 Mediates Lateral Root Development to Improve NUE

Many components of the BR biosynthesis or signaling pathway have been targeted in agricultural domestication programs, highlighting their potential for crop improvement [25, 26, 27, 28, 29, 30]. However, their relationship with N utilization remains largely unclear, impeding the development of sustainable agricultural practices aimed at improving NUE. N and BR share overlapping roles in regulating plant architecture: N deficiency in cereals results in dwarfism, compact plant stature, reduced tillering, and small vegetative organs, which mirrors BR‐deficient phenotypes. Emerging evidences indicate that N‐mediated plant architectural plasticity is largely dependent on BR signaling. For example, natural variation in *DWARF1* (*DWF1*) and *BSK3* contribute to divergent root length responses to mild N deficiency among different *Arabidopsis* ecotypes [12, 31], and *OsTCP19* determines N sensitivity of tiller number in rice natural populations [14]. Our study further reveals the OsGSK2‐OsTCP19 module negatively regulates nitrate‐induced lateral root development in seedling stage and grain yield under field conditions (Figure S6A,E–H), offering a novel molecular target for optimizing NUE in rice breeding.

## 4 Methods

### 4.1 Plant Materials

The rice (*Oryza sativa* L.) varieties Zhonghua 11 (ZH11) and Taichung 65 (TC65) were used in this study. The BR‐related mutants including *bri1* (referred to *d61‐2*), *d11*, *osgsk2*, *osgsk1,2,3,4*, and *dlt* as well as *OsGSK2*‐overexpressing (*GO*) plants were identified in previous studies [32, 33]. The previously identified *ostcp19* mutant and *OsTCP19*‐overexpressing plants were developed in ZH11 background [14]. *osgsk2/35S‐OX1* and *osgsk2/35S‐OX2* lines were generated by crossing *osgsk2* with the *OsTCP19*‐overexpressing plants driven by *CaMV 35S* promoter. *GO/ostcp19* were generated by crossing *GO* with *ostcp19* plants.

### 4.2 Plant Cultivation Conditions

Field trials were performed during the rice cultivation season in 2024 at the experimental station of the Institute of Genetics and Developmental Biology in Beijing. Urea was used as the sole N source, with 50 kg/ha applied for low‐N conditions and 150 kg/ha for moderate‐N conditions.

Hydroponic culture of rice seedlings was carried out in a growth chamber (for 10‐day‐old seedlings) or greenhouse (for 45‐day‐old seedlings) under conditions as previously described [14]. A modified Kimura B solution was used for cultivation, which including 0.37 mM CaCl_2_·2H_2_O, 0.18 mM KH_2_PO_4_, 0.09 mM K_2_SO_4_, 0.55 mM MgSO_4_·7H_2_O, 1.6 mM Na_2_SiO_3_·9H_2_O, 46.2 µM H_3_BO_3_, 0.32 µM CuSO_4_·5H_2_O, 9.14 µM MnCl_2_·4H_2_O, 0.55 µM Na_2_MoO_4_·2H_2_O, 0.76 µM ZnSO_4_·7H_2_O, and 20 µM Fe(II)‐EDTA, pH 5.8. KNO_3_ was used for the sole N source for all the hydroponic cultivation. The nutrient solution was replaced every day for seedlings grown for 10 days or once per week for seedlings grown for 45 days.

### 4.3 Construction of Vectors

To generate the different *OsTCP19*‐overexpressing lines, the coding sequence of *OsTCP19* was amplified from ZH11 and then inserted into *pCAMBIA2300‐35S‐ocs* to produce *35S‐OX1* and *35S‐OX2* lines, *pCAMBIA2300‐35S‐GFP‐ocs* to develop *35S‐OsTCP19‐GFP* lines, and *pCAMBIA1300‐35S‐FLAG‐ocs* vector to produce *35S‐OsTCP19‐FLAG* lines, respectively. In addition, site‐directed mutagenesis by PCR was performed to generate different phosphorylation site mutations in the *OsTCP19* coding sequence, which were then cloned into *pCAMBIA2300‐35S‐GFP‐ocs* to develop *2A‐GFP* and *2D‐GFP* lines, or *pCAMBIA1300‐35S‐FLAG‐ocs* vector to produce *2A_OX* and *2D_OX* lines, respectively.

To generate constructs of *TCP19‐nLUC*, and *GSK2‐cLUC*, the coding sequences of *OsTCP19* and *OsGSK2* were amplified and cloned into *pCAMBIA1300–nLUC* and *pCAMBIA1300‐cLUC*, respectively. Besides, the *OsGSK2* coding sequence was inserted into *pCAMBIA2300‐35S‐GFP‐ocs* for *GSK2‐GFP* construct. For constructs of recombinant protein expression, *OsTCP19* coding sequence was amplified and inserted into pMAL‐C2X for MBP‐TCP19, *OsGSK2* coding sequence was amplified and cloned into pGEX‐4T‐3 for GSK2‐GST.

To generate vectors for transcriptional activity assay, the promoter sequences of OsTCP19‐targeting genes were amplified and cloned into *pGreenII‐0800‐LUC*. Primers used for the construction of these vectors are listed in Table S2.

### 4.4 Lamina Inclination Assay

The lamina inclination assay was modified from a previous study [18]. Uniformly germinated seeds were grown in hydroponic culture of different nitrate concentrations (with or without hormone treatment) for 10 days. The angles between the third leaf blade and sheath were measured for lamina joint inclination using ImageJ software.

### 4.5 Exogenous BL and BRZ Treatment

BL (Sigma) and BRZ (TCI) were dissolved in DMSO as a stock solution and then diluted to different concentrations in the modified Kimura B solution. For lamina inclination measurement and shoot/root phenotyping, rice seedlings grown in hydroponic culture were treated with different BL concentrations or 5 µm BRZ for 10 days. For biomass analysis, rice seedlings were treated with or without 10^−9^ m BL for 45 days.

### 4.6 Root Phenotyping

Seminal root, the first emerging root during seed germination, which shows similar growth trend with other crown roots [34], was selected for the lateral root phenotyping. Seminal roots from 10‐day‐old rice seedlings were sampled, soaked in 75% ethanol, and pictured. Lateral root length and lateral root number of seminal roots were measured using ImageJ software from 12 plants.

### 4.7 Short‐Term Nitrate Induction Assay

Rice seedlings were cultured in the modified Kimura B solution with 0.25 mm (NH_4_)_2_SO_4_ for 1 month in a growth chamber described above. Before nitrate induction, the seedlings were pretreated with continuous light conditions for 48 h and then transferred to the same nutrient solution with 5 mm KNO_3_ or KCl. Roots of rice seedlings were harvested at different time points for gene expression analyses. For BR or BRZ treatment, seedlings were treated with10^−9^ m BL or 5 µm BRZ 12 h ahead of the nitrate induction.

### 4.8 RNA Isolation and RT‐qPCR

Total RNA was extracted from roots using TRIzol reagent (Invitrogen) and reversely transcribed with ReverTra Ace qPCR RT Master Mix (Toyobo) following the manufacturer's instructions. qPCR was performed using the SYBR Green Real‐Time PCR Master Mix kit (Toyobo) with three independent biological replicates. The rice *ACTIN 1* gene was used as the internal reference and primers for qPCR are listed in Table S2.

### 4.9 OsTCP19 Protein Stability Assay in Transgenic Rice

To analyze OsTCP19 protein stability in response to nitrate stimulus, transgenic plants of *35S‐OsTCP19‐GFP* were cultured in modified Kimura B solution containing 0.25 mm (NH_4_)_2_SO_4_ for 1 month. After 48‐h continuous light treatment, rice seedlings were transferred to the same solution with 5 mm KNO_3_ or 5 mm KCl. For BRZ treatment, seedlings were treated with 5 µm BRZ 12 h ahead of the nitrate induction. Total protein was extracted using IP buffer (50 mm Tris‐HCl, pH 7.5, 100 mm NaCl, 1 mm EDTA, 0.25% Triton X‐100, 0.25% NP‐40, 1 × protease inhibitor cocktail, and 10 µm µG132). Immunoblot analysis was performed using anti‐GFP antibody (TransGen, HT801). To compare the protein stability of different OsTCP19 variants, independent transgenic lines (*35S‐OsTCP19‐FLAG*, *35S‐OsTCP19^S141A, T289A^‐FLAG*, and *35S‐OsTCP19^S141D, T289D^‐FLAG*) with comparable expression levels of *OsTCP19* were selected, respectively. Total protein was extracted and immunoblot analysis was performed using anti‐FLAG (Sigma, F1804) antibody. Anti‐Actin antibody (Immunoway, YM3034) served as a protein loading control.

### 4.10 Cell‐Free Degradation Assay

Cell‐free degradation assay was conducted according to previous study [35]. Total proteins were extracted from wild‐type seedlings with extraction buffer containing 5 mm Tris–HCl pH 7.5, 10 mm NaCl, and 10 mm MgCl_2_. recombinant proteins of MBP‐TCP19, MBP‐TCP19^2A^, and MBP‐TCP19^2D^ were incubated with total protein extracts (200 ng purified protein in 250 µL extracts per reaction) at 37°C for 0, 15, and 30 min, respectively. The reactions were stopped by 2 × SDS loading buffer and heated at 95°C for 5 min, separated by SDS‐PAGE, and analyzed by immunoblotting using anti‐MBP (NEB, E8032S) and anti‐Actin (Immunoway, YM3034) antibodies.

### 4.11 Subcellular Localization Assay

To analyze the subcellular localization of OsTCP19 in roots, germinated seeds of *35S‐OsTCP19‐GFP* lines were grown in modified Kimura B solution without N for 5 days, subjected to either 2.5 mm KNO_3_ or 10^−9^ m BL treatment for 2 h, and then fluorescence signals were observed using laser confocal microscope (Carl Zeiss, LSM 980). To detect the subcellular localization of different OsTCP19 variants in roots, germinated seeds from transgenic plants including *35S‐OsTCP19‐GFP*, *35S‐OsTCP19^S141A, T289A^‐GFP*, and *35S‐OsTCP19^S141D, T289D^‐GFP* were grown in modified Kimura B solution without N for 5 days, and the GFP fluorescence signals were observed in the same manner.

### 4.12 LCI Assays

*Agrobacterium tumefaciens* cells carrying *TCP19‐nLUC* and *GSK2‐cLUC* constructs were infiltrated into tobacco leaves. Then the plants were cultured at 22°C for 48 h with 16‐h light/8‐h dark photoperiod. Before detecting luminescence, the infiltrated leaves were sprayed with 1 mm luciferin and kept in darkness for 3 min, and images were captured using a low‐light‐cooled CCD imaging apparatus (NightOWL II LB983).

### 4.13 Co‐IP Assays

Total protein was extracted from infiltrated tobacco leaves with IP buffer containing 50 mm Tris‐HCl pH 7.5, 100 mm NaCl, 1 mm EDTA, 0.25% Triton X‐100, 0.25% NP‐40, 1× protease inhibitor cocktail, and 10 µm µG132. The supernatant was incubated with 20 µL anti‐GFP magnetic agarose beads (Chromotek, gta‐20) for 2 h at 4°C, and then the beads were washed six times with IP buffer. The precipitated proteins were eluted with 2 × SDS loading buffer and heated at 95°C for 5 min, separated by SDS‐PAGE and analyzed by immunoblotting using anti‐GFP (TransGen, HT801) or anti‐FLAG (Sigma, F1804) antibodies.

### 4.14 Phosphorylation Assays

For in vitro protein phosphorylation assays, recombinant proteins of GSK2‐GST and MBP‐TCP19 were expressed in *Escherichia coli strain* BL21 and purified as described previously [14, 33]. Then, different recombinant proteins of GST/GSK2‐GST and MBP/MBP‐TCP19 were incubated in reaction buffer (40 mm Tris‐HCl pH 7.4, 15 mm MgCl_2_, 1 mm DTT, with or without 1 mm ATP) at 37°C for 1 h. The reactions were stopped by 2 × SDS loading buffer and heated at 95°C for 5 min. Samples were separated by normal or Phos‐tag SDS‐PAGE and analyzed by immunoblotting using anti‐MBP (NEB, E8032S) and anti‐GST (Huaxingbio, HX1807) antibodies.

For phosphorylation assay in rice, total proteins were extracted using Co‐IP buffer, OsTCP19‐FLAG was immunoprecipitated (Sigma, M8823), treated with or without 𝜆PP (lambda protein phosphatase) (NEB, P0753) at 30°C for 30 min, reactions were stopped by 5 × SDS loading buffer and heated at 95°C for 5 min, separated by SDS‐PAGE (with Phos‐tag) and analyzed by immunoblotting using anti‐FLAG (Sigma, F1804) and anti‐HSP (Beijing Protein Innovation, AbM51099) antibodies.

### 4.15 Mass Spectrometry Analysis

After the in vitro kinase assay, the samples were separated by normal SDS‐PAGE and digested with trypsin. The peptides were resuspended in 0.1% FA and analyzed by LTQ Orbitrap Elite mass spectrometer (ThermoFisher Scientific) coupled online to an Easy‐nLC 1000 (Thermo Fisher Scientific) in the data‐dependent mode. The peptides were separated by reverse phase LC with a 150 µm (ID) × 250 mm (length) analytical column packed with C18 particles of 1.9 µm diameter. The mobile phases for the LC contains buffer A (0.1% FA) and buffer B (100% ACN, 0.1% FA), and a non‐linear gradient of buffer B from 3%–30% for 90 min was used for the separation. Precursor ions were measured in the Orbitrap analyzer at 240,000 resolution (at 400 m/z) and a target value of 106 ions. The twenty most intense ions from each MS scan were isolated, fragmented, and measured in the linear ion trap. The CID normalized collision energy was set to 35.

### 4.16 ^15^N Accumulation Assay

Rice seedlings were cultivated in modified Kimura B solution containing different nitrate concentrations with or without hormone treatment for 10 days, the nutrient solution was renewed once a day. Before ^15^N‐KNO_3_ accumulation assay, seedlings were pretreated with the same solution for 3 h and then transferred to modified Kimura B solution containing different ^15^N‐KNO_3_ concentrations for ^15^N labeling. After 3‐h treatment, roots of the seedlings were rinsed with 0.1 mm CaSO_4_ and deionized water. Then shoots and roots were collected together, dried at 70°C to a constant weight, and ground to a fine powder. The ^15^N content was determined using an isotope ratio mass spectrometer coupled with an elemental analyzer (Thermo Finnigan Delta Plus XP; Flash EA 1112).

### 4.17 Electrophoretic Mobility Shift Assay (EMSA)

Binding reactions of biotin‐labelled probes and MBP/MBP‐TCP19/MBP‐TCP19^2A^/MBP‐TCP19^2D^ proteins were performed using the LightShift Chemiluminescent EMSA kit (Thermo Fisher Scientific, 20148) according to the manufacturer's instructions. Afterwards, the samples were separated by native PAGE in 0.5 × TBE running buffer for 1 h at room temperature, transferred to nylon membrane at 380 mA for 30 min, crosslinked at 150 mJ/cm^2^, and then the biotin‐labeled probes were detected by chemiluminescence. The probe sequences are listed in Table S2.

### 4.18 Transcriptional Activity Assay in Rice Protoplasts

The transcriptional activity assay of OsTCP19 to different targets was performed using a dual reporter system including firefly luciferase (LUC) and Renilla luciferase (REN). *Firefly luciferase* reporter gene driven by promoters of different target genes were used as reporters. *pCAMBIA2300‐35S‐ocs*, *pCAMBIA2300‐35S::TCP19‐ocs*, *pCAMBIA2300‐35S::TCP19^2A^‐ocs*, *pCAMBIA2300‐35S::TCP19^2D^‐ocs* were used as effectors. Different reporters, effectors and *pCAMBIA2300‐35S::GSK2‐GFP‐ocs* were co‐transfected into nitrate‐free rice protoplasts and incubated in W5 solution for 6 h. The samples were then treated with 5 mm KNO_3_ or KCl for 2 h. Activities of LUC and REN were detected using a Dual‐Luciferase Reporter Assay System kit (Promega, E1960). The ratio of LUC/REN was indexed as the relative transcriptional activity.

## Author Contributions

Y.L., W.L., X.M., and X.W. performed most of the experiments, X.H. and Y.W. carried out the mass spectrum analysis, Y.S., X.L., H.T., B.H., and W.Z. performed some of the experiments. Y.L. and C.C. designed the research, wrote the manuscript, and supervised the project.

## Conflicts of Interest

The authors declare no conflicts of interest.

## Supporting information




**Supporting File**: advs74856‐sup‐0001‐SuppMat.docx.

## Data Availability

The data that supports the findings of this study are available in the supplementary material of this article.
