# Supplementary material for: OsGSK2‐OsTCP19 Module Integrates Nitrogen and Brassinosteroid Signaling to Regulate Nitrogen Utilization and Root Growth in Rice
Source: Adv Sci (Weinh). 2026 Mar 15;13(30):e74856. doi: 10.1002/advs.74856 (PMC13248756; doi:10.1002/advs.74856)
Supplement: Supplementary file 1 — Supporting File: advs74856‐sup‐0001‐SuppMat.docx. [file ADVS-13-e74856-s001.docx]

| 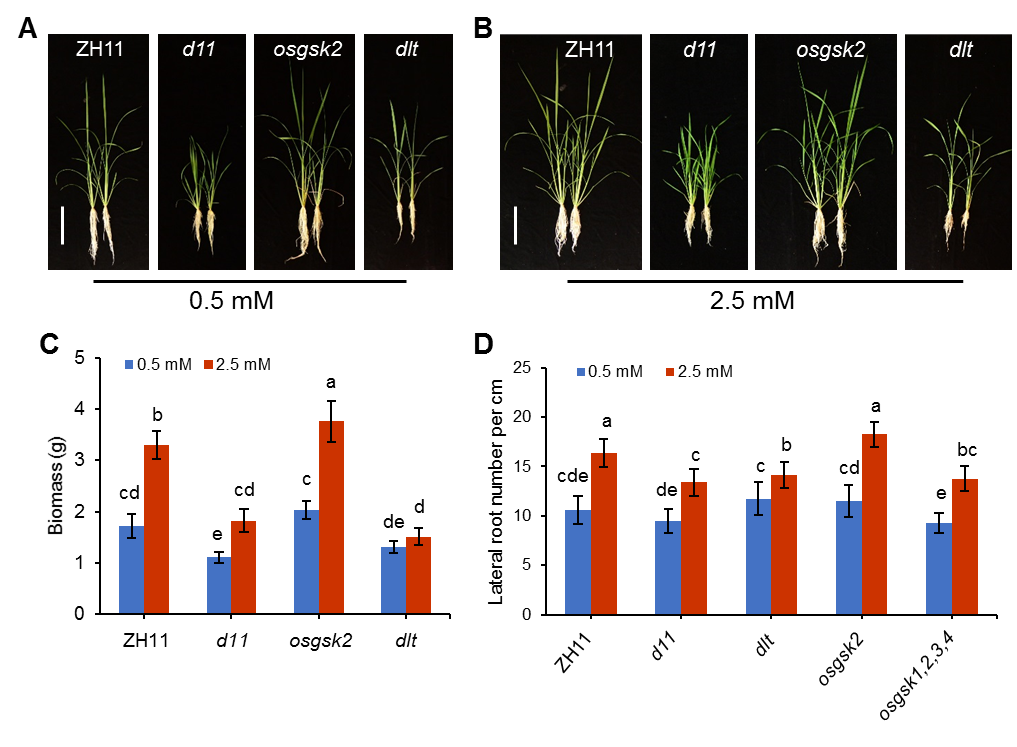 |
| --- |


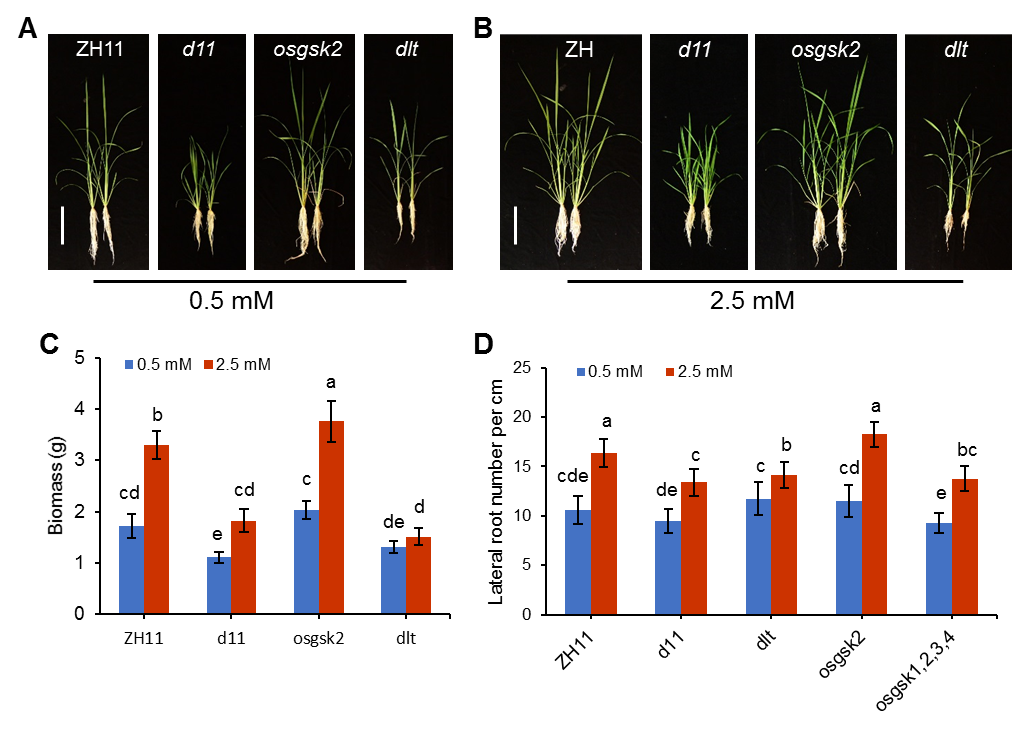


**Supplemental Figure 1 | Nitrate-activated plant growth depends on BR response in rice. A**, **B**, Phenotype of wild-type ZH11, BR-deficient (*d11*), BR-insensitive (*dlt*), and BR-hypersensitive (*osgsk2*) mutants grown under low- (0.5 mM) and high-nitrate (2.5 mM) conditions for 45 days. Bars = 12 cm. **C**, Biomass of wild type (WT) and BR-related mutants of **A** and **B**. Data are mean ± s.d. (n = 10 plants). **D**, Lateral root number per cm of ZH11 and BR-related mutants. Data are mean ± s.d. (n = 12 plants). The different letters in **C** and **D** indicate significant differences (*P* < 0.05, one-way ANOVA, Tukey’s HSD test).

| 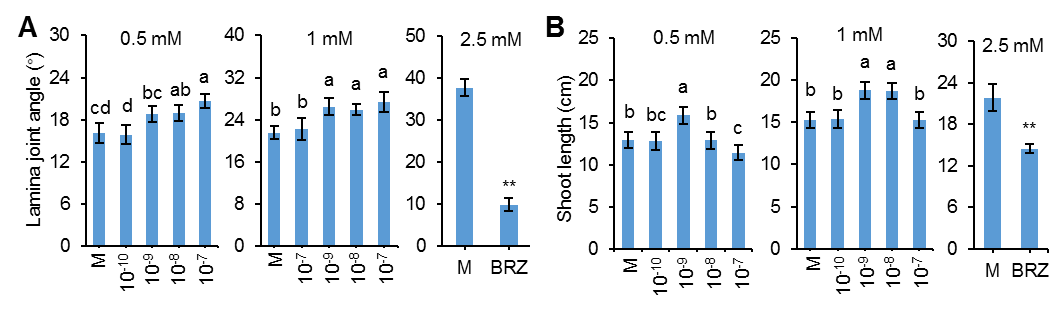 |
| --- |

**Supplemental Figure 2 | Nitrate interacts with BR to regulate rice seedling growth. A**, **B**, Lamina joint angle (**A**) and shoot length (**B**) of rice seedlings grown under different nitrate concentrations (0.5, 1, 2.5 mM) treated with 0 (mock, M), 10^-10^, 10^-9^, 10^-8^, and 10^-7^ M BL. Data are mean ± s.d. (n = 8 plants). Different letters indicate significant differences (*P* < 0.05, one-way ANOVA, Tukey’s HSD test). Asterisks indicate significant differences between mock and BRZ treatment: ***P* < 0.01 (Student’s *t*-test).

| 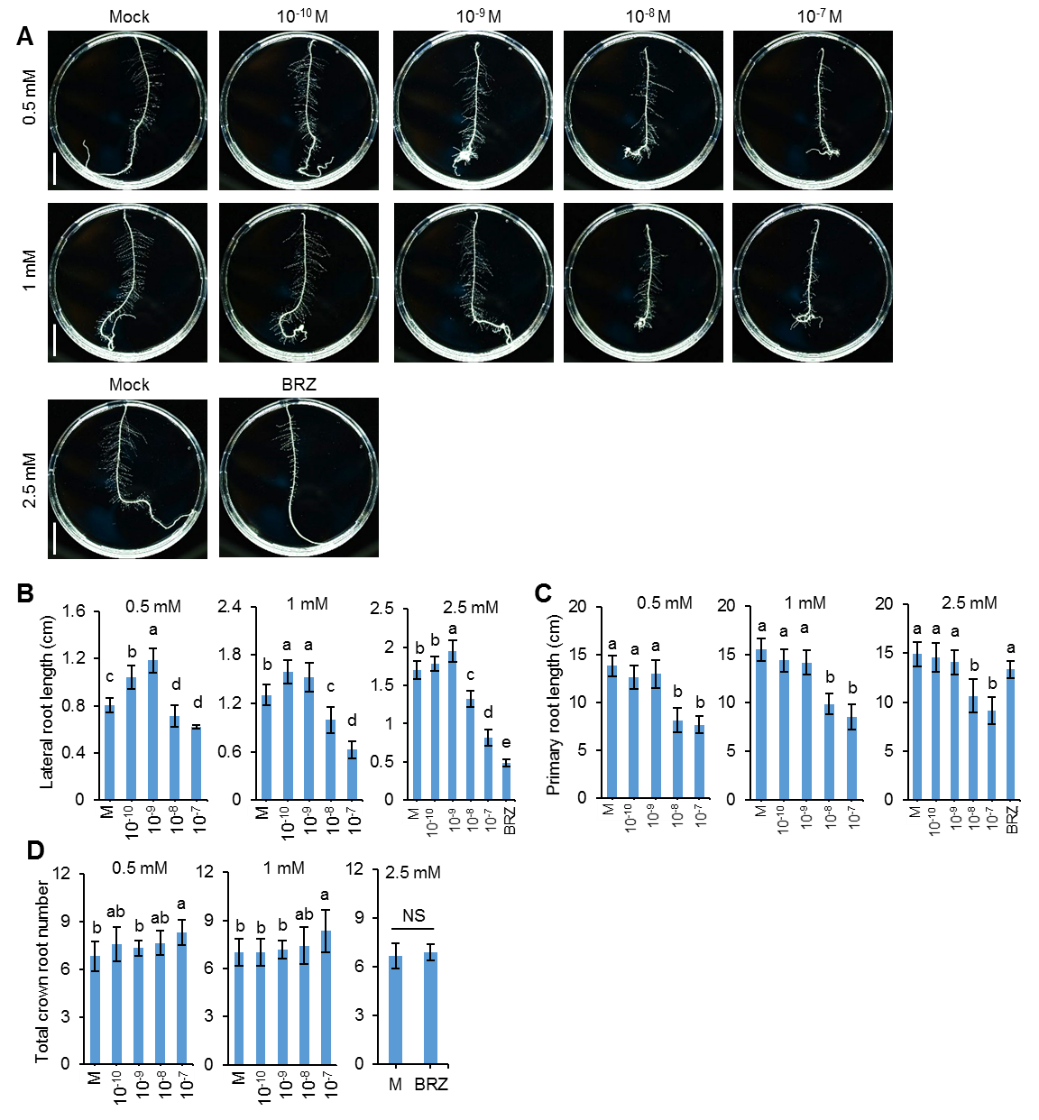 |
| --- |

**Supplemental Figure 3 | BR regulates rice root development in dose-dependent manner which also relies on nitrate concentration. A**, Phenotype of primary roots of ZH11 grown under different nitrate concentrations (0.5, 1, 2.5 mM) treated with 0 (mock, M), 10^-10^, 10^-9^, 10^-8^, and 10^-7^ M BL. Bars = 2 cm. **B**, **C**, Statistical analysis of lateral root length (**B**) and primary root length (**C**) of ZH11. **D**, Total crown root number of ZH11 seedlings grown under conditions from **A**. For **B** - **D**, data are mean ± s.d. (n = 12 plants). Different letters indicate significant differences (*P* < 0.05, one-way ANOVA, Tukey’s HSD test). Asterisks indicate significant differences between mock and BRZ treatment: ***P* < 0.01 (Student’s *t*-test). NS, no significance.

| 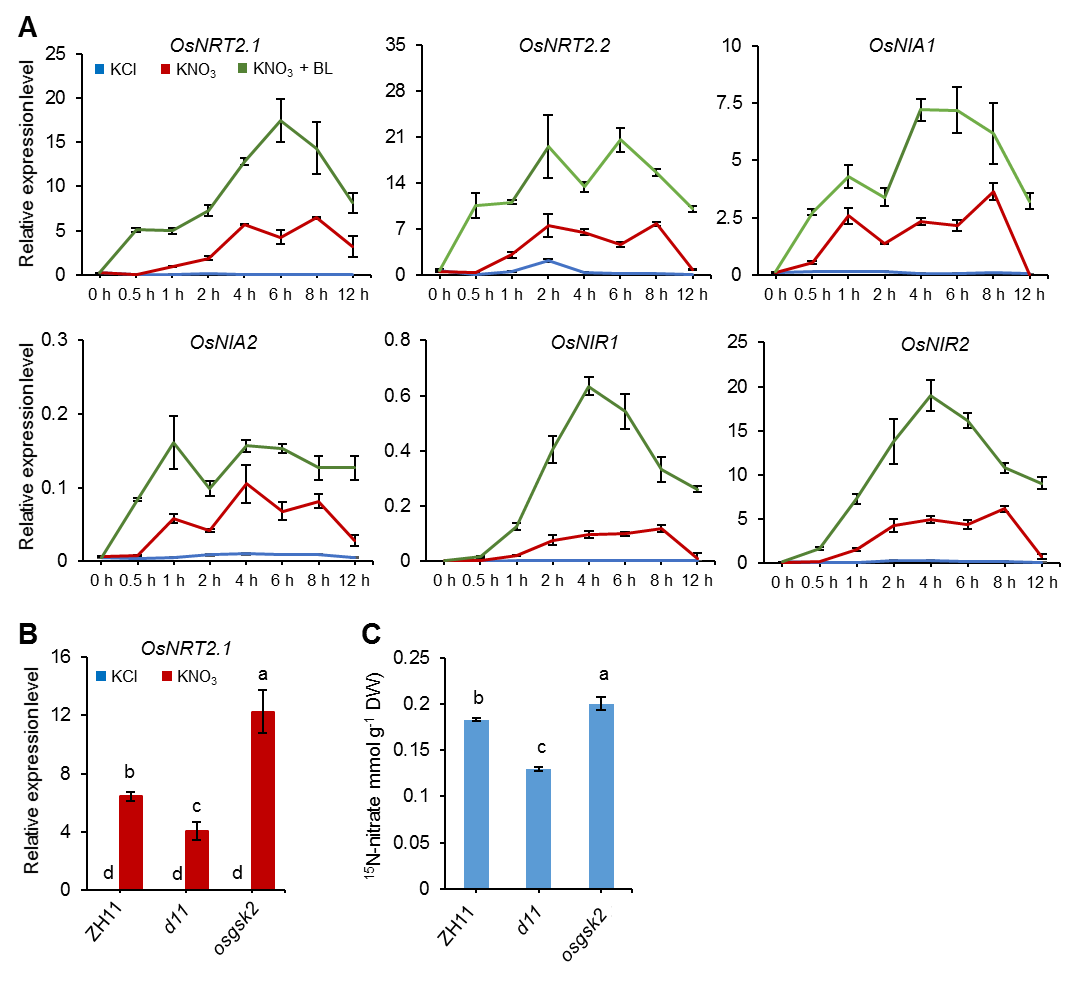 |
| --- |

**Supplemental Figure 4 | BR positively regulates nitrate response and uptake in rice. A**, Nitrate induction (5 m M KNO_3_) assay of nitrate-responsive genes in the roots of ZH11 plants treated with or without 10^-9^ M BL. 21-day-old seedlings were used for nitrate induction. KCl was used for the negative control. **B**, Nitrate induction assay of nitrate-responsive genes in the roots of ZH11, *d11*, and *osgsk2* plants (5 mM KNO_3_, 8 h). Values are the means ± s.d. n = 3 biologically independent samples. **C**, ^15^N-nitrate accumulation in rice seedlings of ZH11, *d11*, and *osgsk2* mutants. Data are mean ± s.d. (n = 3 biologically independent samples). Different letters indicate significant differences (*P* < 0.05, one-way ANOVA, Tukey’s HSD test).

| 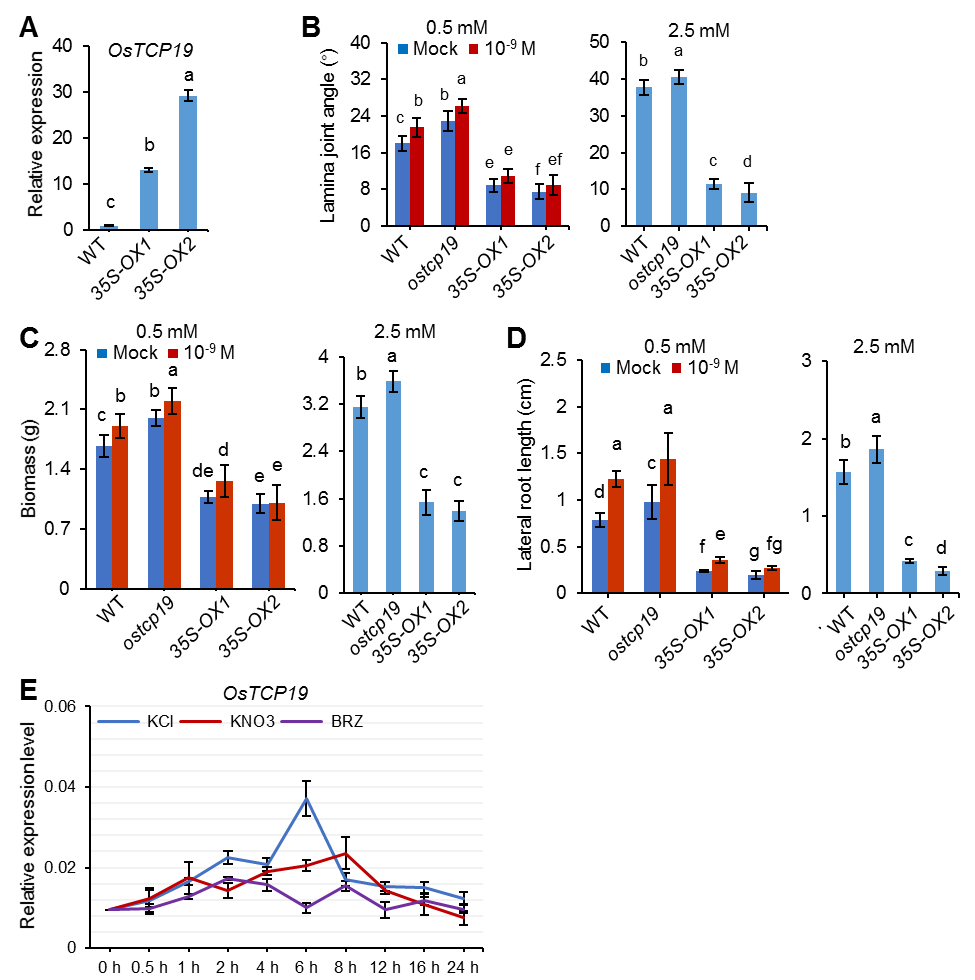 |
| --- |

**Supplemental Figure 5 | *OsTCP19* negatively participates in nitrate/BR-stimulated shoot and root development in rice. A**, Relative expression level of WT and OsTCP19-overexpressing lines (*35S-OX1* and *35S-OX2*). Values are the means ± s.d. (n = 3 biologically independent samples). **B**, Lamina joint angle of rice seedlings including WT, *ostcp19* mutant, *35S-OX*, and *35S-OX2* plants grown under different conditions. **C,** Statistical analysis of biomass (dry weight) of WT, *ostcp19* mutant, *35S-OX*, and *35S-OX2* plants. **D,** Statistical analysis of lateral root length of WT, *ostcp19* mutant, *35S-OX1* and *35S-OX2* plants. **E**, Nitrate induction assay of *OsTCP19* in the roots of ZH11 plants treated with or without 5 μM brassinazole (BRZ). KCl was used for the negative control. Values are the means ± s.d. (n = 3 biologically independent samples). For **B** and **C**, data are mean ± s.d. (n = 12 plants). Different letters indicate significant differences (*P* < 0.05, one-way ANOVA, Tukey’s HSD test).

| 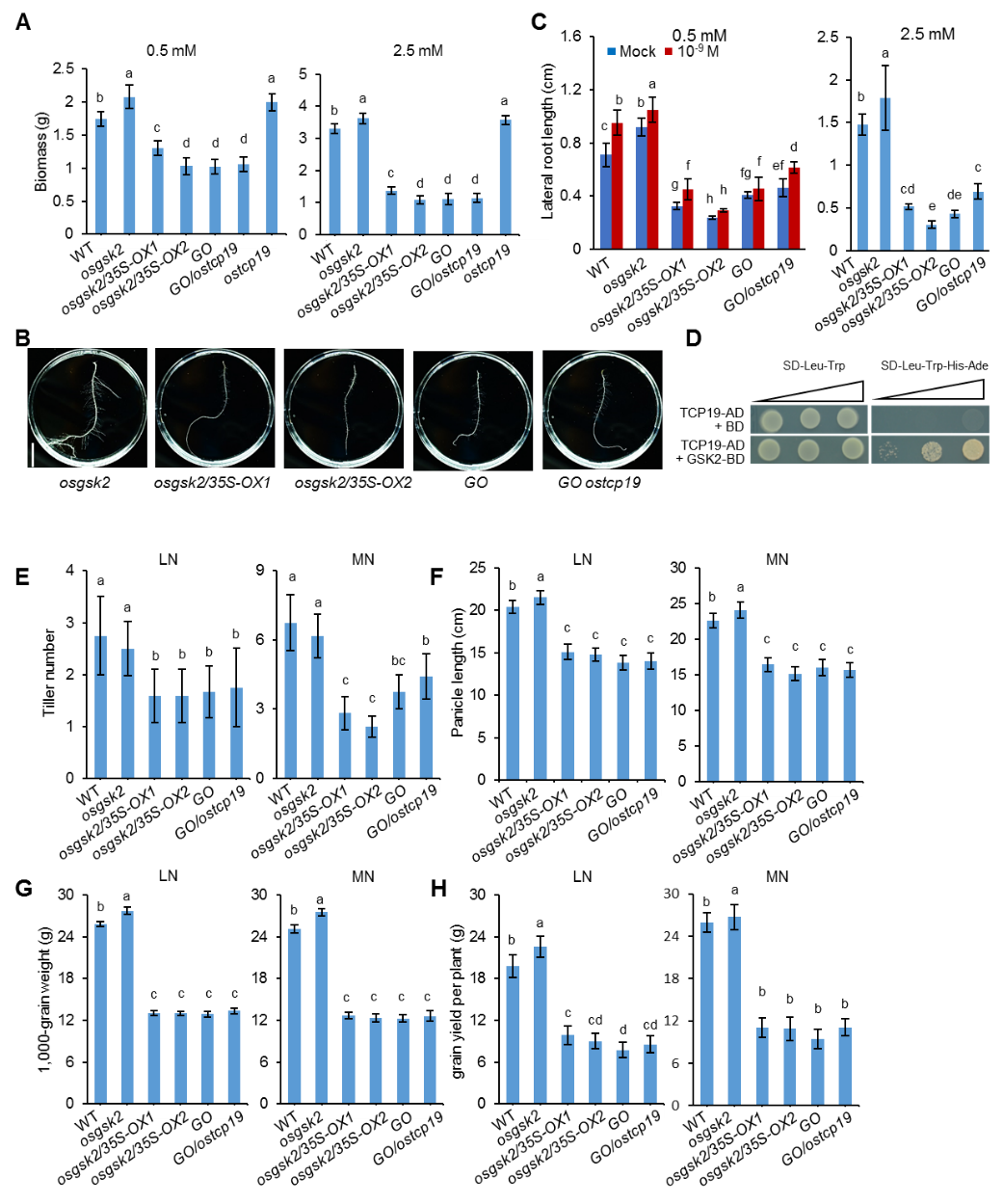 |
| --- |

**Supplemental Figure 6 | *OsTCP19* interacts with and genetically acts downstream of *OsGSK2*. A,** Statistical analysis of biomass (dry weight) of *osgsk2*, *OsTCP19*-overexpressing lines under *osgsk2* background (*osgsk2/35S-OX1* and *osgsk2/35S-OX2*), *GO*, *GO/ostcp19*, and *ostcp19* plants. **B**, Phenotype of primary roots of *osgsk2*, *OsTCP19*-overexpressing lines under *osgsk2* background (*osgsk2/35S-OX1* and *osgsk2/35S-OX2*), *GO*, and *GO/ostcp19* plants grown under high-nitrate conditions. Bar = 2 cm. **C**, Statistical analysis of lateral root length of ZH11, *osgsk2*, *OsTCP19*-overexpressing lines under *osgsk2* background, *GO*, and *GO/ostcp19* plants grown under low-nitrate, low-nitrate and 10^-9^ M BL, and high-nitrate conditions. Data are mean ± s.d. (n = 12 plants). **D**, Yeast two-hybrid assay showing the interaction between OsGSK2 and OsTCP19. OsTCP19 fused to the GAL4 activation domain (AD) and OsGSK2 fused to the binding domain (BD) were co-transformed. Triangles represent yeast clones grown on the SD plates with different dilutions (10^0^, 10^-1^, 10^-2^). **E-H**, Agronomic traits of different genetic lines under low-nitrogen (LN) and moderate-nitrogen (MN) conditions including tiller number (**E**), panicle length (**F**), 1,000-grain weight (**G**), and grain yield per plant (**H**). Different letters indicate significant differences (*P* < 0.05, one-way ANOVA, Tukey’s HSD test). ).

| 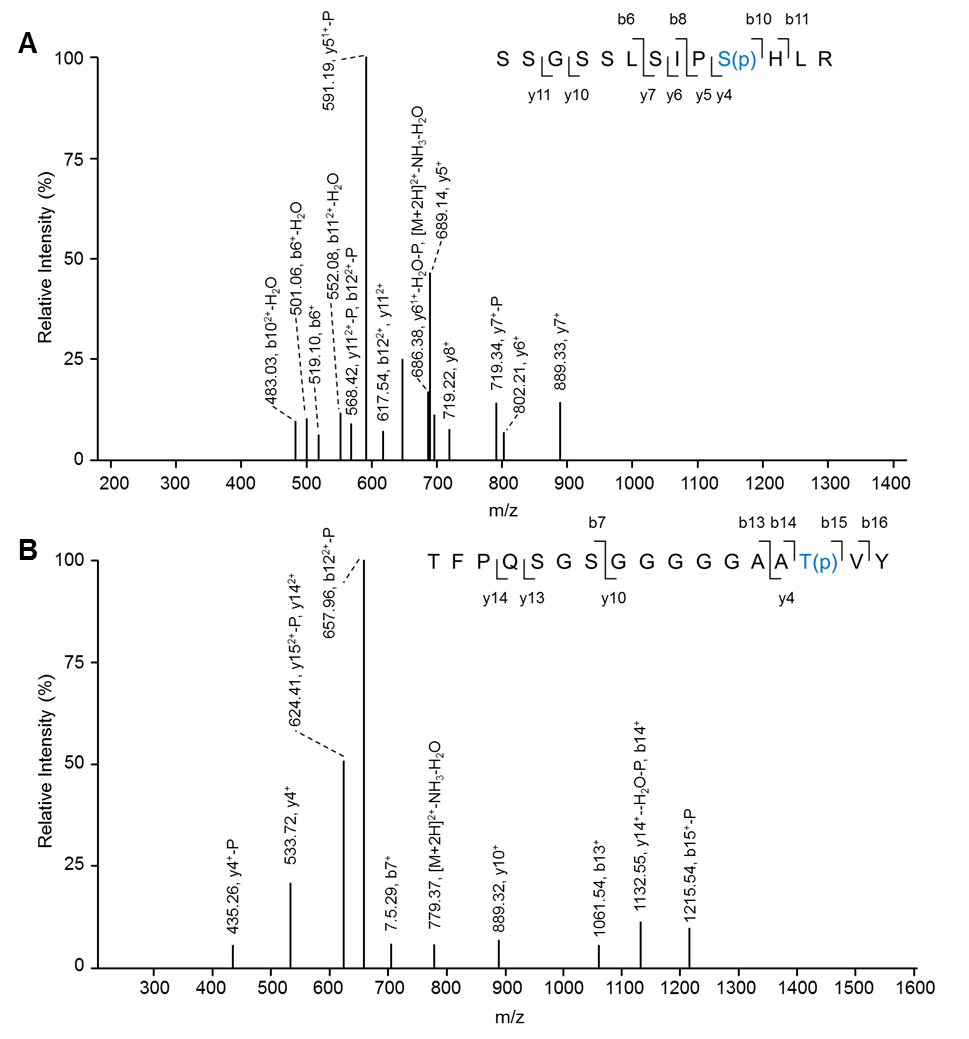 |
| --- |

**Supplemental Figure 7 | OsGSK2 phosphorylates OsTCP19 at Ser141 and Thr289 identified by mass spectrum analyses. A**, **B**, Phosphorylated peptides containing Ser141 site (**A**) and Thr289 site (**B**) were identified by mass spectrum analysis after in vitro phosphorylation reaction. The Ser141 and Thr289 residues were highlighted.

| 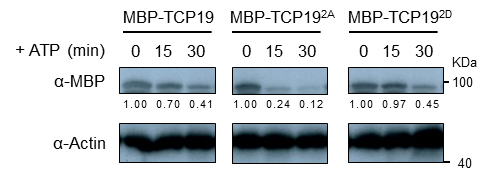 |
| --- |

**Supplemental Figure 8 | Phosphorylation of OsTCP19 stabilizes its protein level.** Cell-free degradation assays showing the degradation rates of different variants of OsTCP19. Recombinant proteins of MBP-TCP19, MBP-TCP19^2A^, and MBP-TCP19^2D^ were incubated with total protein extracts of the wild-type ZH11 seedlings for indicated period. Actin was used as loading control. Each band was quantified by ImageJ.

| 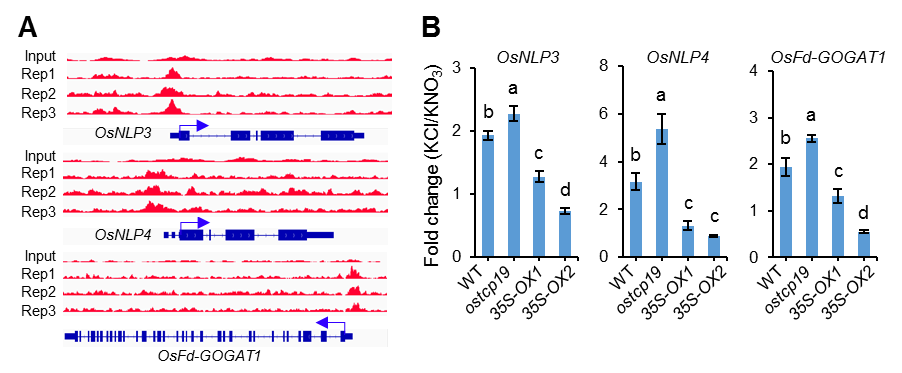 |
| --- |

**Supplemental Figure 9 | OsTCP19 targets to nitrate-responsive genes of *OsNLP3*, *OsNLP4*, and *OsFd-GOGAT1*. A**, OsTCP19-binding profile of nitrate-responsive genes including *OsNLP3*, *OsNLP4*, and *OsFd-GOGAT1*. **B**, Nitrate induction assay of nitrate-responsive genes in the roots of WT, *ostcp19*, and *OsTCP19*-overexpressing lines (5 mM KNO_3_, 6 h). Values are the means ± s.d. (n = 3 biologically independent samples). Different letters indicate significant differences (*P* < 0.05, one-way ANOVA, Tukey’s HSD test).

| 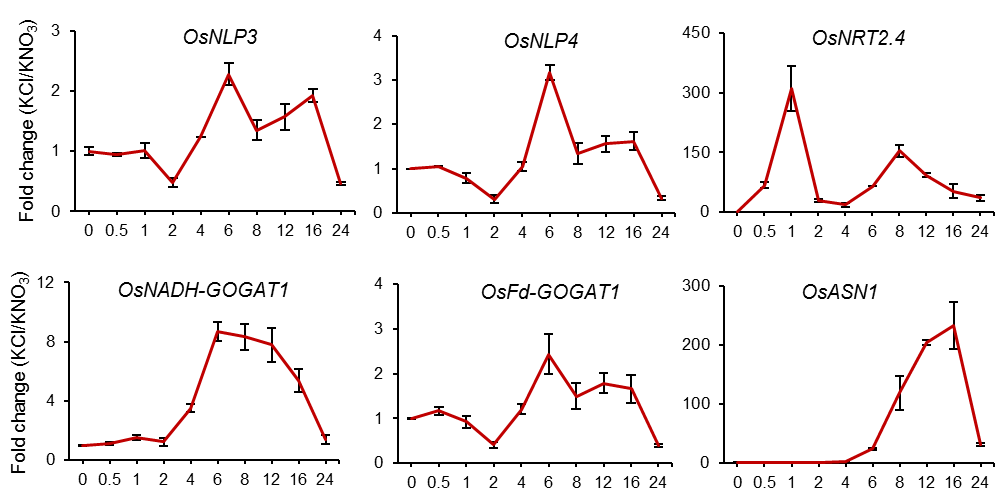 |
| --- |

**Supplemental Figure 10 | Nitrate induction assay of N-related genes targeted by OsTCP19 in the roots of ZH11 within 24 h**. Values are the means ± s.d. (n = 3 biologically independent samples).

| 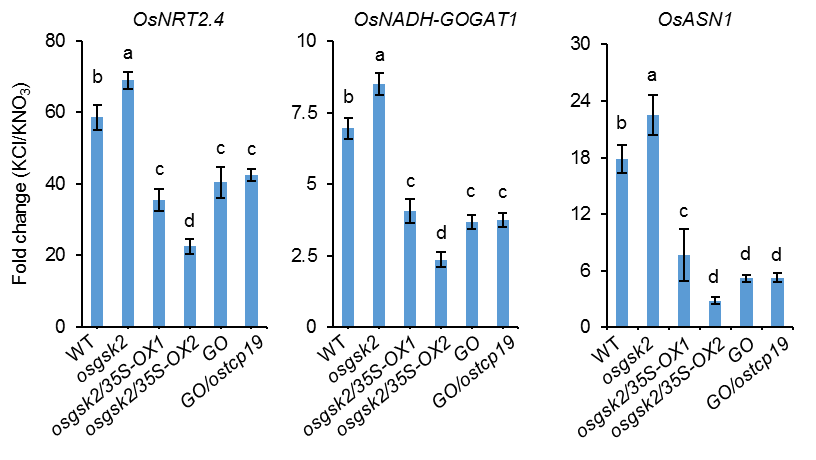 |
| --- |

**Supplemental Figure 11 | *OsGSK2* negatively regulates the expression of nitrate-responsive gens partially dependent on *OsTCP19*.** Nitrate induction assay of nitrate-responsive genes including *OsNRT2.4*, *OsNADH-GOGAT1*, and *OsASN1* in the roots of different genetic lines (5 mM KNO_3_, 6 h). Values are the means ± s.d. n = 3 biologically independent samples. Different letters indicate significant differences (*P* < 0.05, one-way ANOVA, Tukey’s HSD test).

| 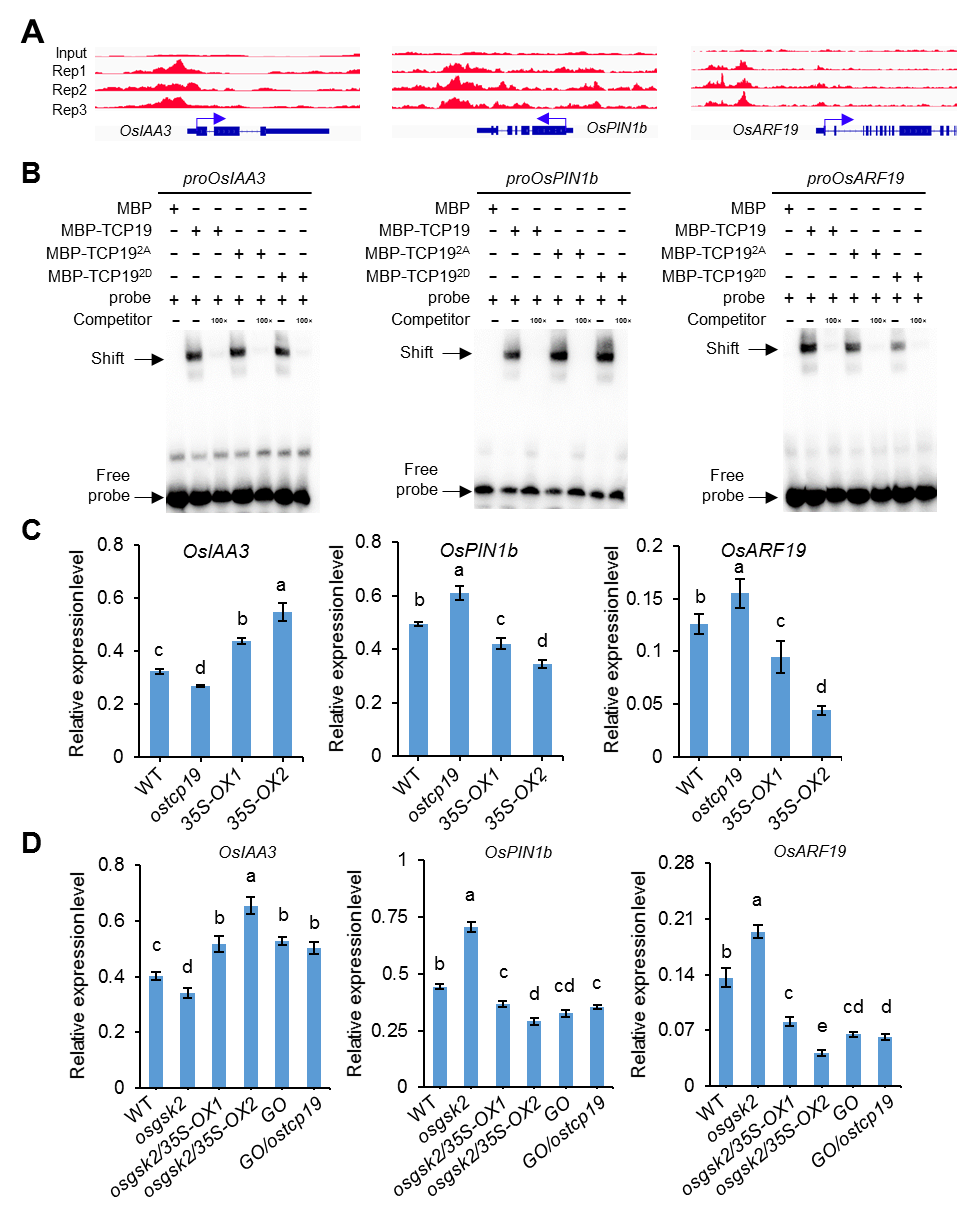 |
| --- |

**Supplemental Figure 12 | OsTCP19 targets to root-development genes downstream of *OsGSK2*. A**, OsTCP19-binding profile of root-development genes including *OsIAA3*, *OsPIN1b*, and *OsARF19*. **B**, Different OsTCP19 variants including MBP-TCP19, MBP-TCP19^2A^, and MBP-TCP19^2D^ can directly bind to the promoters including *OsIAA3*, *OsPIN1b*, and *OsARF19* in EMSA. 100-fold unlabeled wild-type probes were used for competition. **C**, Relative expression level of root-development genes in the roots of WT, *ostcp19*, and *OsTCP19*-overexpressing lines. **D**, Relative expression level of root-development genes in the roots of different genetic lines. Values are the means ± s.d. (n = 3 biologically independent samples). Different letters indicate significant differences (*P* < 0.05, one-way ANOVA, Tukey’s HSD test).

| 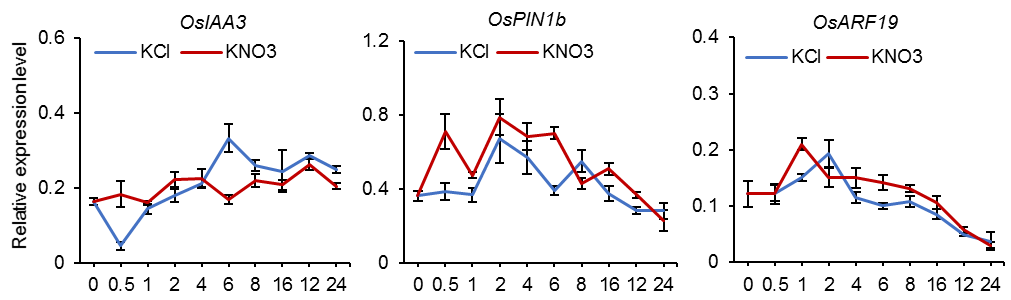 |
| --- |

**Supplemental Figure 13 | Nitrate induction assay of *OsIAA3*, *OsPIN1b*, and *OsARF19* in the roots of ZH11 plants**. KCl was used for the negative control. Values are the means ± s.d. (n = 3 biologically independent samples). 0 to 24 indicate the induction time (hours).

**Supplemental Table 1. Root development-related genes identified from OsTCP19 ChIP-seq datasets.**

| **Locus_ID** | **Start** | **End** | **Length** | **Count** | **Score** | **Strand** | **Annotation** | **Distance** |
| --- | --- | --- | --- | --- | --- | --- | --- | --- |
| LOC_Os12g40900 | 25325123 | 25325519 | 397 | 2 | 0 | + | OsIAA31_-_Auxin-responsive_Aux/IAA_gene_family_member,expressed | -31 |
| LOC_Os01g49000 | 28136014 | 28136341 | 328 | 2 | 0 | + | katanin_p60_ATPase-containing_subunit,putative,expressed | -27 |
| LOC_Os07g37400 | 22413396 | 22413570 | 175 | 1 | 0 | - | OsFBX257_-_F-box_domain_containing_protein,expressed | -326 |
| LOC_Os02g50550 | 30871374 | 30871528 | 155 | 1 | 0 | - | dynamin,putative,expressed | -204 |
| LOC_Os02g50960 | 31158074 | 31158482 | 409 | 2 | 0 | - | auxin_efflux_carrier_component,putative,expressed | 422 |
| LOC_Os05g03430 | 1426660 | 1426922 | 263 | 2 | 0 | + | ATSIZ1/SIZ1,putative,expressed | -8 |
| LOC_Os04g57610 | 34288407 | 34288699 | 293 | 1 | 0 | - | auxin_response_factor,putative,expressed | -2539 |
| LOC_Os01g58420 | 33761946 | 33762826 | 881 | 2 | 0 | + | AP2_domain_containing_protein,expressed | -4154 |
| LOC_Os01g58420 | 33762625 | 33762898 | 274 | 1 | 0 | + | AP2_domain_containing_protein,expressed | -4082 |
| LOC_Os01g58420 | 33764923 | 33765073 | 151 | 1 | 0 | + | AP2_domain_containing_protein,expressed | -1907 |
| LOC_Os01g58420 | 33765264 | 33766098 | 835 | 3 | 0 | + | AP2_domain_containing_protein,expressed | -882 |
| LOC_Os01g58420 | 33768424 | 33769288 | 865 | 3 | 0 | + | AP2_domain_containing_protein,expressed | 340 |
| LOC_Os01g04330 | 1932104 | 1932694 | 591 | 1 | 0 | - | OsCML16_-_Calmodulin-related_calcium_sensor_protein,expressed | -201 |
| LOC_Os01g04330 | 1932374 | 1932635 | 262 | 1 | 0 | - | OsCML16_-_Calmodulin-related_calcium_sensor_protein,expressed | -471 |
| LOC_Os02g04160 | 1819191 | 1819369 | 179 | 1 | 0 | - | transcription_elongation_factor_1,putative,expressed | -98 |
| LOC_Os04g38720 | 22996754 | 22997055 | 302 | 2 | 0 | + | no_apical_meristem_protein,putative,expressed | 15 |
| LOC_Os01g36720 | 20388447 | 20389026 | 580 | 1 | 0 | - | transporter,major_facilitator_family,putative,expressed | 0 |
| LOC_Os08g06110 | 3358355 | 3358575 | 221 | 1 | 0 | + | MYB_family_transcription_factor,putative,expressed | -7156 |
| LOC_Os08g06110 | 3358360 | 3359003 | 644 | 1 | 0 | + | MYB_family_transcription_factor,putative,expressed | -6728 |
| LOC_Os08g05840 | 3136393 | 3136534 | 142 | 1 | 0 | + | DNA_topoisomerase_1,putative,expressed | -211 |
| LOC_Os06g03600 | 1395683 | 1396877 | 1195 | 3 | 0 | - | transcriptional_corepressor_SEUSS,putative,expressed | 0 |
| LOC_Os10g05690 | 2853976 | 2854461 | 486 | 1 | 0 | - | transmembrane_amino_acid_transporter_protein,putative,expressed | 0 |
| LOC_Os02g46780 | 28558485 | 28558822 | 338 | 2 | 0 | - | MYB_family_transcription_factor,putative,expressed | 0 |
| LOC_Os02g41510 | 24883268 | 24883406 | 139 | 1 | 0 | - | MYB_family_transcription_factor,putative,expressed | -3335 |
| LOC_Os08g31580 | 19546765 | 19547480 | 716 | 3 | 0 | + | ethylene-responsive_transcription_factor,putative,expressed | 0 |
| LOC_Os01g63770 | 36999922 | 37000118 | 197 | 1 | 0 | + | transmembrane_amino_acid_transporter_protein,putative,expressed | 0 |
| LOC_Os01g63770 | 37002732 | 37003101 | 370 | 2 | 0 | + | transmembrane_amino_acid_transporter_protein,putative,expressed | 0 |
| LOC_Os01g63770 | 37003316 | 37003596 | 281 | 2 | 0 | + | transmembrane_amino_acid_transporter_protein,putative,expressed | 0 |
| LOC_Os04g50770 | 30029028 | 30029505 | 478 | 2 | 0 | - | MYB_family_transcription_factor,putative,expressed | 0 |
| LOC_Os04g50770 | 30029039 | 30029371 | 333 | 1 | 0 | - | MYB_family_transcription_factor,putative,expressed | 0 |
| LOC_Os02g51860 | 31768526 | 31769671 | 1146 | 3 | 0 | - | dehydration_response_related_protein,putative,expressed | 0 |
| LOC_Os06g02480 | 857938 | 858444 | 507 | 2 | 0 | - | expressed_protein | 0 |
| LOC_Os10g39030 | 20808112 | 20808591 | 480 | 3 | 0 | - | homeobox_domain_containing_protein,expressed | -1453 |
| LOC_Os09g36160 | 20840694 | 20840966 | 273 | 1 | 0 | - | LRP1,putative,expressed | -403 |
| LOC_Os10g34520 | 18409878 | 18410135 | 258 | 1 | 0 | + | mitosis_protein_dim1,putative,expressed | -1372 |
| LOC_Os04g58960 | 35075097 | 35075619 | 523 | 2 | 0 | - | regulator_of_chromosome_condensation,putative,expressed | -186 |
| LOC_Os01g62244 | 36024652 | 36025061 | 410 | 3 | 0 | - | ubiquitin-conjugating_enzyme,putative,expressed | -1184 |
| LOC_Os11g44810 | 27118637 | 27119148 | 512 | 3 | 0 | - | auxin-repressed_protein,putative,expressed | -629 |
| LOC_Os09g37710 | 21734992 | 21735290 | 299 | 1 | 0 | + | NIN,putative,expressed | -151 |
| LOC_Os05g32140 | 18736667 | 18736972 | 306 | 1 | 0 | + | NAD_dependent_epimerase/dehydratase_family_protein,putative,expressed | -1626 |
| LOC_Os06g40080 | 23857874 | 23858414 | 541 | 3 | 0 | - | heme_oxygenase_1,putative,expressed | 0 |
| LOC_Os06g41990 | 25210733 | 25210887 | 155 | 1 | 0 | - | M16_domain_containing_zinc_peptidase,putative,expressed | -200 |
| LOC_Os02g02890 | 1115439 | 1116087 | 649 | 3 | 0 | + | peptidyl-prolyl_cis-trans_isomerase,putative,expressed | 0 |
| LOC_Os02g02890 | 1118642 | 1119365 | 724 | 3 | 0 | + | peptidyl-prolyl_cis-trans_isomerase,putative,expressed | 1507 |
| LOC_Os06g48950 | 29650555 | 29651640 | 1086 | 3 | 0 | + | auxin_response_factor_19,putative,expressed | -5330 |
| LOC_Os06g48950 | 29650588 | 29651258 | 671 | 1 | 0 | + | auxin_response_factor_19,putative,expressed | -5712 |
| LOC_Os06g48950 | 29652279 | 29653053 | 775 | 3 | 0 | + | auxin_response_factor_19,putative,expressed | -3917 |
| LOC_Os06g48950 | 29656888 | 29657244 | 357 | 1 | 0 | + | auxin_response_factor_19,putative,expressed | 0 |
| LOC_Os03g50520 | 28846403 | 28846969 | 567 | 3 | 0 | + | dynamin_family_protein,putative,expressed | 0 |
| LOC_Os02g04680 | 2105504 | 2105722 | 219 | 1 | 0 | + | OsSPL3_-_SBP-box_gene_family_member,expressed | -5 |
| LOC_Os04g21350 | 12063834 | 12064151 | 318 | 1 | 0 | - | flowering_promoting_factor-like_1,putative,expressed | -7 |
| LOC_Os04g46580 | 27613728 | 27614727 | 1000 | 3 | 0 | + | OsSPL7_-_SBP-box_gene_family_member,expressed | -50 |
| LOC_Os01g60960 | 35264317 | 35264538 | 222 | 2 | 0 | + | DUF260_domain_containing_protein,putative,expressed | 4870 |
| LOC_Os03g03150 | 1334945 | 1335086 | 142 | 1 | 0 | + | WD_repeat-containing_protein,putative,expressed | 3736 |
| LOC_Os05g32270 | 18811634 | 18811887 | 254 | 1 | 0 | + | AP2_domain_containing_protein,expressed | -504 |
| LOC_Os05g32270 | 18811726 | 18812085 | 360 | 1 | 0 | + | AP2_domain_containing_protein,expressed | -306 |
| LOC_Os07g39630 | 23752631 | 23753077 | 447 | 1 | 0 | + | calcium-binding_protein,putative,expressed | -32 |
| LOC_Os07g39630 | 23752883 | 23753618 | 736 | 1 | 0 | + | calcium-binding_protein,putative,expressed | 0 |
| LOC_Os08g04620 | 2305491 | 2305741 | 251 | 1 | 0 | + | MSP_domain_containing_protein,putative,expressed | -69 |
| LOC_Os10g28040 | 14556153 | 14556368 | 216 | 1 | 0 | - | histone_acetyltransferase_GCN5,putative,expressed | -607 |
| LOC_Os05g51510 | 29541806 | 29542195 | 390 | 1 | 0 | - | protein_phosphatase_2C,putative,expressed | 0 |
| LOC_Os03g31750 | 18161431 | 18162049 | 619 | 3 | 0 | - | pyruvate,phosphate_dikinase,chloroplast_precursor,putative,expressed | -1305 |
| LOC_Os08g13920 | 8319613 | 8319816 | 204 | 1 | 0 | + | glycosyl_hydrolases_family_16,putative,expressed | -8051 |
| LOC_Os08g13920 | 8322949 | 8323248 | 300 | 1 | 0 | + | glycosyl_hydrolases_family_16,putative,expressed | -4619 |
| LOC_Os11g36440 | 21485018 | 21486055 | 1038 | 3 | 0 | + | amine_oxidase,flavin-containing,domain_containing_protein,expressed | 0 |
| LOC_Os06g44010 | 26509177 | 26509347 | 171 | 1 | 0 | - | WRKY28,expressed | 573 |
| LOC_Os01g18240 | 10223074 | 10223311 | 238 | 1 | 0 | - | MYB_family_transcription_factor,putative,expressed | -3379 |
| LOC_Os01g16430 | 9329326 | 9329578 | 253 | 2 | 0 | - | cysteine_proteinase_inhibitor_precursor_protein,putative,expressed | 0 |
| LOC_Os10g31910 | 16758609 | 16759184 | 576 | 3 | 0 | - | pectate_lyase_precursor,putative,expressed | 0 |
| LOC_Os04g52479 | 31214503 | 31215268 | 766 | 3 | 0 | + | peptidase,trypsin-like_serine_and_cysteine_proteases,putative,expressed | 0 |
| LOC_Os09g16510 | 10122635 | 10122787 | 153 | 1 | 0 | + | WRKY74,expressed | -6039 |
| LOC_Os09g16510 | 10122812 | 10123183 | 372 | 2 | 0 | + | WRKY74,expressed | -5643 |
| LOC_Os04g32030 | 19201629 | 19201907 | 279 | 1 | 0 | + | heavy_metal-associated_domain_containing_protein,expressed | 9238 |
| LOC_Os04g32030 | 19205381 | 19205691 | 311 | 2 | 0 | + | heavy_metal-associated_domain_containing_protein,expressed | 12990 |
| LOC_Os06g49010 | 29704573 | 29704769 | 197 | 1 | 0 | - | OsSPL12_-_SBP-box_gene_family_member,expressed | 0 |
| LOC_Os01g03630 | 1489891 | 1490568 | 678 | 2 | 0 | - | multicopper_oxidase_domain_containing_protein,expressed | 0 |

**Supplemental Table 2. Primers (5'-3') used in this study.**

| **Primers for qRT-PCR** | |
| --- | --- |
| Actin1-qRT F | ACCATTGGTGCTGAGCGTTT |
| Actin1-qRT R | CGCAGCTTCCATTCCTATGAA |
| OsTCP19-qRT F | GACAGTGTACCGTGGCGT |
| OsTCP19-qRT R | CGCCGGGAAGTTCATGAAAT |
| OsNRT2.1-qRT F | CTTGTTGCAAACGGTGATGA |
| OsNRT2.1-qRT R | GCCTCTCCCTTATTATACCTCCG |
| OsNRT2.2-qRT F | CGGAGCACGCCTAATTAAGAG |
| OsNRT2.2-qRT R | CTCCATGACGACATACTCTAGATA |
| OsNIA1-qRT F | CCTGGAGAAGATGGGCTAT |
| OsNIA1-qRT R | GCACAACCATCCATCAATC |
| OsNIA2-qRT F | TGTACCAGGTCATCCAGTCG |
| OsNIA2-qRT R | CGATGACGTACCACACCTTG |
| OsNIR1-qRT F | CTGCCTCACCAAGGACAG |
| OsNIR1-qRT R | TTCCTACTCCTCGTCCTCCT |
| OsNIR2-qRT F | GAACGAGGAGTAGGAGCACA |
| OsNIR2-qRT R | GGGCTACAAGATCAAACCAA |
| OsNLP3-qRT F | CATTGTCAGGTTCCGCTTCC |
| OsNLP3-qRT R | ATGCATTCTTCCAAGTCCGC |
| OsNLP4-qRT F | TGGCTGCTAGTCCAACAACA |
| OsNLP4-qRT R | GACCTGCATCTGATTGTGGC |
| OsNRT2.4-qRT F | AAAGGTCGCTGGGCGTGGTG |
| OsNRT2.4-qRT R | CCTGGACCCGCTGAAGAAGAG |
| OsNADH-GOGAT1-qRT F | GAAACAGGCAAACTTTTCAAGAC |
| OsNADH-GOGAT1-qRT R | GCATTTCACCATGCAAATCA |
| OsFd-GOGAT1-qRT F | GCATACTTGTGAAGCACCGAAGTG |
| OsFd-GOGAT1-qRT R | CTGCAAATAGCAACCTAGCGTCAG |
| OsASN1-qRT F | ACCGCATGATATTTGAGAGG |
| OsASN1-qRT R | CTGCTCCACCCACTCGAT |
| OsIAA3-qRT F | CGACGTCCCATTCGAGATGT |
| OsIAA3-qRT R | TTGCTCCTAGGCCTCTTGCTT |
| OsPIN1b-qRT F | CCTGACAACCAGCCATGTTA |
| OsPIN1b-qRT R | CTCGTTTGACTCCCTTCCAA |
| OsARF19-qRT F | ATCTCTTAAAGAGGGCTATGCC |
| OsARF19-qRT R | GTTGCTCAATGATCGAAGGTAC |
| **Primers for vector construction** | |
| 35S-OsTCP19 F | GGGTACCCGGGGATCCATGGATGTCACCGGAGACGG |
| 35S-OsTCP19 R | ATGCCTGCAGGTCGACCTACGAGTCGCTGGCGCTCA |
| OsTCP19-GFP F | TTTGGAGAGGACAGGGTACCATGGATGTCACCGGAGACGG |
| OsTCP19-GFP R | TAGTGTCGACTCTAGACGAGTCGCTGGCGCTCAT |
| OsTCP19-FLAG F | CACGGGGGACTCTAGAATGGATGTCACCGGAGACGG |
| OsTCP19-FLAG R | TGTAGTCCATGTCGACCGAGTCGCTGGCGCTCAT |
| OsGSK2-GFP F | TTTGGAGAGGACAGGGTACCATGGACCAGCCGGCGCCG |
| OsGSK2-GFP R | TAGTGTCGACTCTAGAGCTCCCAGTATTGAAGAA |
| OsTCP19-nLUC F | CGGGGGACGAGCTCGGTACCATGGATGTCACCGGAGACGG |
| OsTCP19-nLUC R | ACGAGATCTGGTCGACCGAGTCGCTGGCGCTCA |
| OsGSK2-cLUC F | ACGCGTCCCGGGGCGGTACCATGGACCAGCCGGCGCCGGC |
| OsGSK2-cLUC R | TACGAACGAAAGCTCTGCAGTTAGCTCCCAGTATTGAAGA |
| OsTCP19-MBP F | AAGGATTTCAGAATTCATGGATGTCACCGGAGACGG |
| OsTCP19-MBP R | TTGCCTGCAGGTCGACCTACGAGTCGCTGGCGCTCAT |
| OsGSK2-GST F | GGTTCCGCGTGGATCCATGGACCAGCCGGCGCCGGC |
| OsGSK2-GST R | CTCGAGTCGACCCGGGTTAGCTCCCAGTATTGAAGA |
| pGL-proOsNRT2.4 F | CCCCCTCGAGGTCGACCGTAGCCGTGATTGAACCAG |
| pGL-proOsNRT2.4 R | TTGGCGTCTTCCATGGGATGGAGGGGCAGAGCTAG |
| pGL-proOsNADH-GOGAT1 F | CCCCCTCGAGGTCGACATCGAATCCGTCCCTCGTTG |
| pGL-proOsNADH-GOGAT1 R | TTGGCGTCTTCCATGGCGACTCTCCCTCTGCTACCT |
| pGL-proOsASN1 F | CCCCCTCGAGGTCGACATAGATCTGTAGCCCGCTGC |
| pGL-proOsASN1 R | TTGGCGTCTTCCATGGGACGGTGGATGGATGCGTAT |
| pGL-proOsIAA3-qRT F | CCCCCTCGAGGTCGACAACAGTCAAACATGCACCGC |
| pGL-proOsIAA3-qRT R | TTGGCGTCTTCCATGGCGAGCTCCTCCTCCTCTTCT |
| pGL-proOsPIN1b-qRT F | CCCCCTCGAGGTCGACCGTGTTGGACAGAGTCAGCT |
| pGL-proOsPIN1b-qRT R | TTGGCGTCTTCCATGGCCTCTTCCCTCCTCCTCCTC |
| pGL-proOsARF19-qRT F | CCCCCTCGAGGTCGACTGGAGATGTTTTCAGGTGAC |
| pGL-proOsARF19-qRT R | TTGGCGTCTTCCATGGCAGACCAGACAGGCAGACAC |
| **Primers for EMSA** |  |
| OsNRT2.4-probe F | ATGTGGGCCCCACCCTCTCCAGGGCCCACTTGCC |
| OsNRT2.4-probe R | GGCAAGTGGGCCCTGGAGAGGGTGGGGCCCACAT |
| OsNADH-GOGAT1-probe F | CGGAGCGGGGTGGGCCCACCATGGCCACCA |
| OsNADH-GOGAT1-probe R | TGGTGGCCATGGTGGGCCCACCCCGCTCCG |
| OsASN1-probe F | CCCACGGTGCAGGGCTCACCCTCCTGTCCC |
| OsASN1-probe R | GGGACAGGAGGGTGAGCCCTGCACCGTGGG |
| OsIAA3-probe F | GAGGCGTCAGGGGGCCCACGGGGTCGGCCC |
| OsIAA3-probe R | GGGCCGACCCCGTGGGCCCCCTGACGCCTC |
| OsPIN1b-probe F | AAGGGGTTGGTGGCCCTACGTTTGGTGCAG |
| OsPIN1b-probe R | CTGCACCAAACGTAGGGCCACCAACCCCTT |
| OsARF19-probe F | TGGCGATGGCCCCACCGGCCAGGGGCCCAGGGAT |
| OsARF19-probe R | ATCCCTGGGCCCCTGGCCGGTGGGGCCATCGCCA |
